# Trees represent community composition of other plant life-forms, but not their diversity, abundance or responses to fragmentation

**DOI:** 10.1038/s41598-018-29635-9

**Published:** 2018-07-27

**Authors:** Bonifacio O. Pasion, Mareike Roeder, Jiajia Liu, Mika Yasuda, Richard T. Corlett, J. W. Ferry Slik, Kyle W. Tomlinson

**Affiliations:** 1 Center for Integrative Conservation, Xishuangbanna Tropical Botanical Garden, Chinese Academy of Sciences, Menglun, Mengla, Yunnan 666303 China; 2 University of the Chinese Academy of Sciences, Beijing, China; 3 Key Laboratory of Conservation Biology for Endangered Wildlife of the Ministry of Education, College of Life Sciences, Zhejiang University, Hangzhou, China; 4 Birdlife International Tokyo, 4F TM Suidobashi Bldg., 2-14-6 Misaki-cho, Chiyoda-ku, Tokyo 101-0061 Japan; 5 Faculty of Science, Environmental and Life Sciences, Universiti Brunei Darussalam, Gadong, BE1410 Brunei Darussalam

## Abstract

Our understanding of the patterns of plant diversity in tropical forests and their responses to fragmentation are mostly based on tree surveys. But are these patterns and responses representative of other plant life-forms? We sampled trees, lianas, herbs, and ferns in a fragmented tropical forest landscape in South-west China. We compared community types generated by clustering presence-absence data for the non-tree life-forms with those generated for trees. We tested how well measures of tree diversity, density and composition, predicted cognate indices in other life-forms. We compared fragmentation responses, with respect to the three measures, of all four life-forms. Presence-absence data from all life-forms generated three community clusters, with only small differences between classifications, suggesting that tree data identified community types representative of all vascular plant life-forms. Tree species diversity and density indices poorly predicted cognate indices of lianas and ferns, but represented herbs well. However, the slopes of these relationships differed substantially between community types. All life-forms responded to fragmentation variables but their responses did not consistently match with responses of trees. Plot-level tree data can identify vegetation community types, but is poorly representative of the richness and density of other life-forms, and poorly represents forest fragmentation responses for the entire plant community.

## Introduction

Trees, due to their large biomass, play a crucial role in many ecosystems by influencing ecosystem processes such as nutrient cycling^1,2^, carbon storage^3^, and regulation of understory microclimate^4,5^ (e.g. shading, moderating temperature and humidity extremes, reducing precipitation throughfall). In vegetation studies, trees are commonly used to understand ecological correlates of composition, richness and density^6–10^. Other plant life-forms, such as lianas, herbs, and ferns, are less studied, although they contribute substantially to plant richness and density^11–15^. Although inferences about community structure and change are mostly based on trees, whether they adequately represent all other plant life-forms has rarely been tested (e.g.^16,17^). If trees are representative of all plant life-forms, then studying just trees is enough. If not, then other plant life-forms should be measured directly to understand how they respond to ecological drivers. Failing to do so will result in conservation plans and management strategies that address the needs of trees only at the expense of other plant life-forms.

An assessment of whether trees are representative of other plant life-forms in a landscape needs to consider their representativeness at different levels. First, landscapes are likely to support multiple plant communities composed of different species in response to resource gradients across the landscape and these plant communities may differ in their susceptibility to environmental change^18,19^. Therefore, at the first level, it is important to assess whether tree data can be used to effectively identify the component communities in a landscape. This can be evaluated by testing whether the community types identified by tree data are representative of the community types identified by the data for other life-forms. Plant community classifications are most-often based on tree data only (e.g.^6,7,20^), but it has rarely been shown that these classifications are representative of all life-forms in the community^13,14,21^. Second, having correctly identified the community types in the landscape, it is important to recognise that community composition, richness and density will change within individual communities based on more local environmental drivers. Therefore, it is important to demonstrate that changes in diversity and density of different life-forms within each community can be effectively represented by the changes to diversity and density of trees in those communities.

Third, if trees are to be used as surrogates for whole communities, then it is necessary to demonstrate that changes happening to tree composition, diversity and density in response to environmental change are representative of changes happening to the other life-forms. This is particularly pertinent in the Anthropocene when habitats have been—and continue to be—fragmented by human activities. Forest fragmentation is the process by which a large tract of forest is broken into smaller and more isolated patches^22^ leading to losses of forest species and their replacement by non-forest or non-native species^23,24^. However, most fragmentation studies of plants have concentrated on trees, which might be expected to respond slowly to environmental changes relative to other life-forms because of their long life spans and generation times, and larger size^25–27^. By comparison, rapid responses to fragmentation have been observed in herbs^28,29^, lianas^30,31^, and ferns^32,33^, suggesting that trees may not represent their dynamics well. Studies of multiple plant life-forms provide a more robust understanding of fragmentation impacts on plant communities^34,35^, but they are rare.

In this study, we compared patterns of species composition, diversity, and density, for trees, lianas, herbs, and ferns in 48 permanent sampling plots across a fragmented forest landscape in Xishuangbanna, southwest China. We asked the following questions: (1) Are plant communities, identified by tree data from the plots, representative of communities identified for other plant life-forms? (2) Within community types, does tree data reliably predict the composition, diversity, and density of other plant life-forms? (3) Are the community responses of other life-forms to habitat fragmentation qualitatively similar to those of trees?

## Results

### Community classification

**Figure 1:**
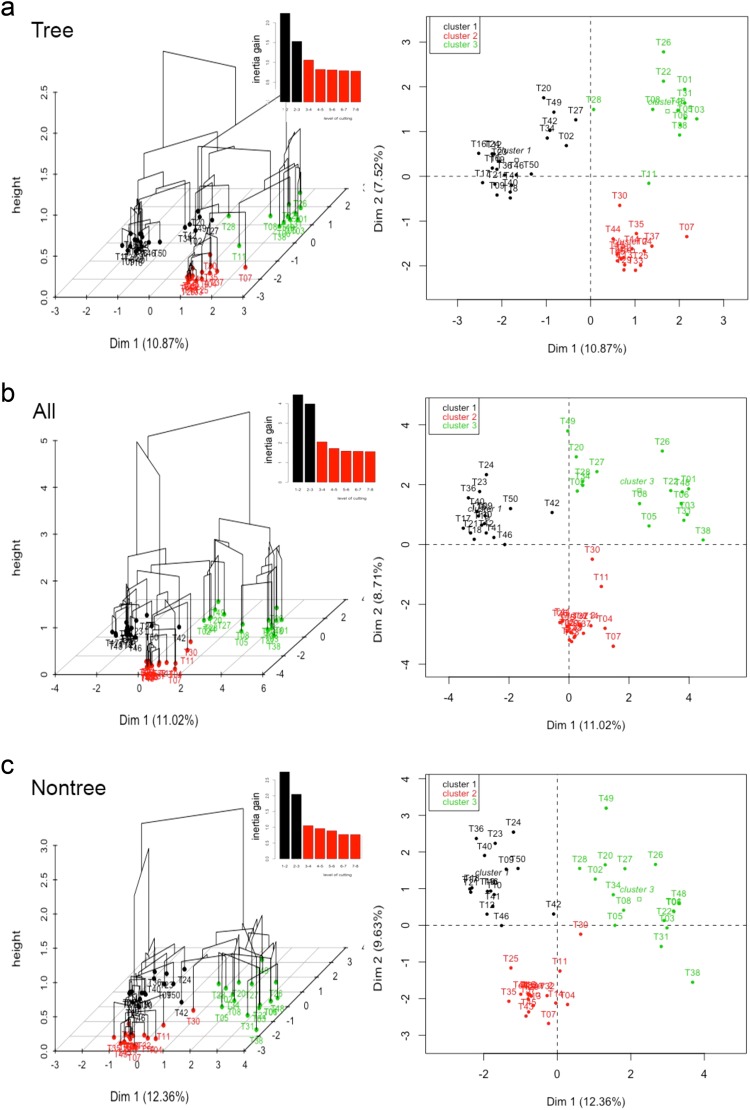
Clusters generated by hierarchical clustering on principal components (HCPC) on the presence-absence plot data from the Xishuangbanna forest fragment plots for (**a**) tree species data only, (**b**) species of all life-forms (“all data”; including trees, lianas, herbs and ferns), (**c**) species of all plant life-forms except trees data (“non-tree data”; including lianas, herbs and ferns). Clusters are first selected using hierarchical clustering with Ward’s criterion and a specified number of clusters. Subsequently individual sites may be reassigned to other groups on the basis of K-means clustering. Figures show clustering with three groups specified. Inertia graphics indicate that inertia is best accounted for by three groups. HCPC results for separate HCPC analyses of liana data, herb data, and fern data, are provided in Supplementary Fig. S2. All data and non-tree data formed identical groups. Tree data differed with non-tree data for six plots, where five lowland plots (T2, T20, T27, T34 and T49) in non-tree data are clustered as montane in tree data and one limestone plot (T11) in non-tree data are clustered as lowland plot in tree data. Data taken from 48 plots in forest fragments in Xishuangbanna, southwest China.

**Figure 2:**
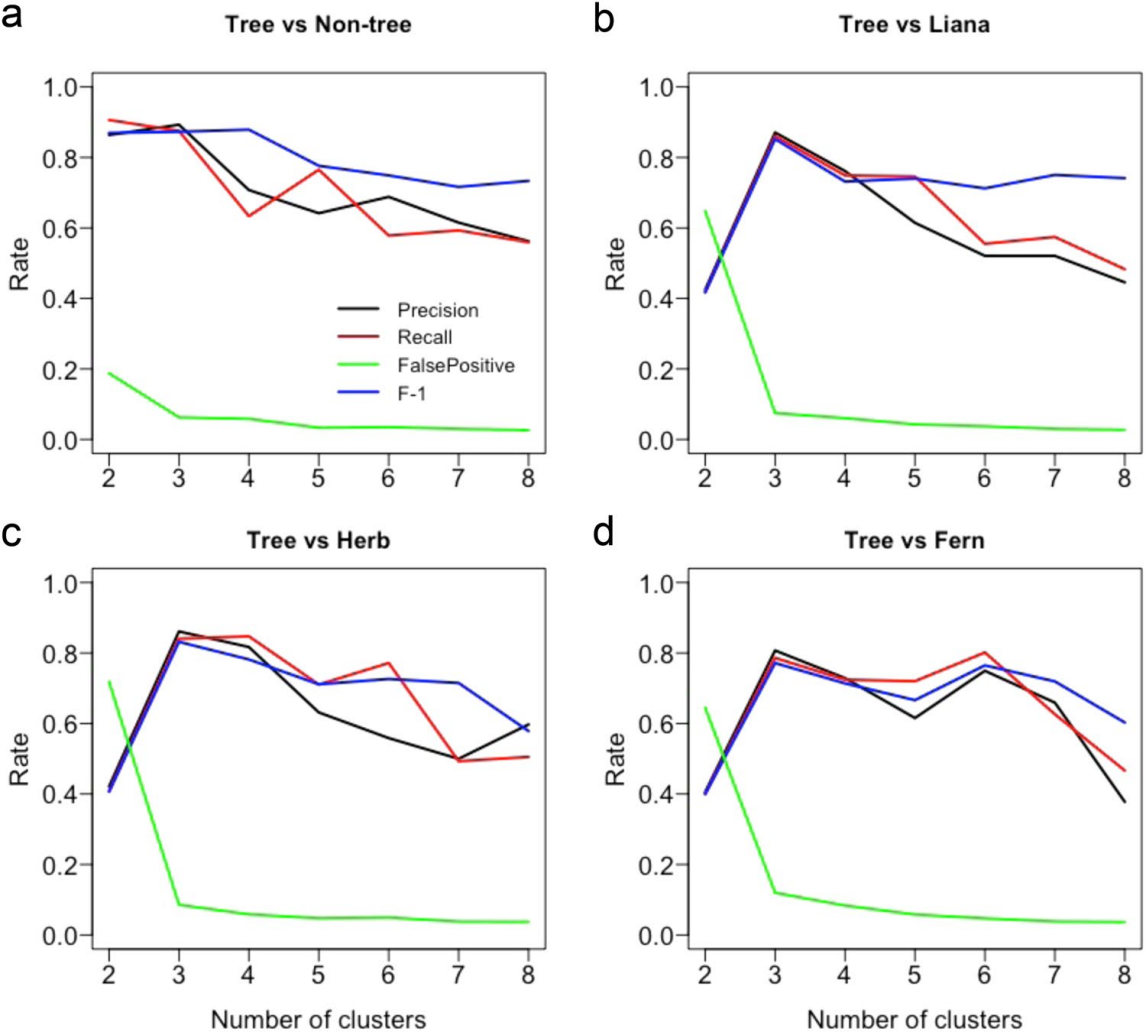
Confusion matrix measures generated when using tree data cluster membership (see Fig. 3) to predict cluster membership generated for (**a**) non-tree data (combined lianas, herbs, ferns), (**b**) liana data, (**c**) herb data and (**d**) fern data. We treat the tree data as the predictor data and the other life-form’s data as the ‘actual’ data. For each data set, plots are assigned to different clusters by HCPC for a specified number of clusters ranging from 2 to 8 clusters. The confusion matrix measures evaluate how well the prediction data (tree data) performs in assigning each data point (our plots) to the correct class as specified by the ‘actual’ data for the same number of clusters generated by predictor and actual data. Using the confusion matrix, we calculated the precision (=true positives/(true positives + false positives), sensitivity (recall) (the true positive rate, = true positives/(predicted positives)), false positives (incorrectly assigned to a group when not an ‘actual’ member of that group), and F1 (the harmonic mean of precision and sensitivity) measures based on those confusion matrices (see ref.^65^ for more details about confusion matrices and measures). Values close to 1 for precision, recall and F-1, and close to 0 for the false positive rate, indicate high agreement between the predictor data and the actual data. The measures indicate that 3 groups give optimal congruence between tree data and the other life-forms. This is turn suggests that 3 community types are present in our study site. Data taken from 48 plots in forest fragments in Xishuangbanna, southwest China.

First, we clustered the plot data for each life-form based on presence-absence data across the plots using hierarchical clustering on principal components (HCPC)^36^, and then assessed whether the clusters generated by the tree data were representative of clusters generated by other life-forms using confusion matrix measures (see *Methods*). We used presence-absence data to make the community classification so that communities were based on member identity not on abundance (the latter is used in subsequent analyses). Figure 1 shows the clustering result for tree data only, non-tree data (all other life-forms combined), and all data (all life-forms combined); Supplementary Fig. S1 shows the clustering results for each of the non-tree life-forms. Simultaneously, the tree data predicted other groups optimally for three clusters as shown using the confusion matrix measures comparing the ability of tree data to predict plot group membership correctly for the other life-forms (Fig. 2). The tree data achieved an F1 value of between 0.77 for fern data up to 0.87 for non-tree data (Supplementary Table S1). The proportion of false positives also declined dramatically from 2 clusters to 3 clusters, and then fell very slowly for larger cluster numbers. Thus the combined information from the HCPC analysis and the confusion matrices analysis suggests that the best-supported cluster number was 3 clusters. Therefore, we assumed the three clusters generated by the all species data (identical to that produced by the non-tree data) were the most representative of community types in our plot data set. The three community types identified are similar to three Asian forest types described in Cao and Zhang^37^, namely: ‘monsoon forest over limestone soils’ (hereafter ‘limestone forest’) (16 plots), ‘tropical seasonal forest’ (hereafter ‘lowland forest’) (16 plots), and ‘evergreen broad-leaved forest’ (hereafter ‘montane forest’) (16 plots). Table 1 provides a summary of the plot classification differences under tree data versus other life-forms. Tree data differed from non-tree data (lianas, herbs and ferns together) for six plots out of 48 (the total number of plots). Tree data differed from liana data for seven plots out of 48, from herb data for eight plots out of 48, and from fern data for 11 plots out of 48.

**Table 1:** Confusion matrix data comparing community type assignments for each sampled plot between tree data and other life-forms (non-tree, liana, herb, and fern data), generated by clustering using HCPC for each life-form group (see *Materials and Methods* for further details).

Life-form		Tree data		
		Limestone	Lowland	Montane
Non-tree data (lianas, herbs, ferns)	Limestone	15	1	0
	Lowland	0	11	5
	Montane	0	0	16
Liana data	Limestone	14	1	0
	Lowland	1	11	5
	Montane	0	0	16
Herb data	Limestone	15	1	2
	Lowland	0	11	5
	Montane	0	0	14
Fern data	Limestone	14	0	3
	Lowland	1	11	6
	Montane	0	1	12

Rows represent assignments under the non-tree data and columns represent assignments under tree data. Diagonal values indicate where assignments of under tree data are in agreement with assignments under other life-forms.

The three community types could be distinguished with respect to limestone/non-limestone soils (as measured by soil pH), elevation, and topography (Supplementary Fig. S2). Limestone forest has a significantly greater pH than the other two community types, and lowland forest was at lower elevation than either limestone forest or montane forest. Accordingly, limestone forest and montane forest were predominantly associated with ridges and slopes whereas lowland forest was predominantly associated with valleys. The plots of the three communities are well-distributed across the range of fragment sizes and distances to forest edge (Supplementary Fig. S3), and therefore the discrimination of the community types is not related to these anthropogenic predictors.

The observed richness and density of each life-form in each of the community types is given in Table S2 (Supplementary Information), and the most abundant species of each life-form associated with each community type is provided in Table S3 (Supplementary Information). Tree and fern richness differed among all community types, with lowland forest > montane forest > limestone forest. Species richness and density of lianas and herbs was significantly higher in lowland forest than in montane forest or limestone forest. The densities of trees in both lowland forest and limestone forest were lower than in montane forest, while the densities of ferns in both montane forest and limestone forest were lower than in lowland forest.

### Life-form diversity and density

**Figure 3:**
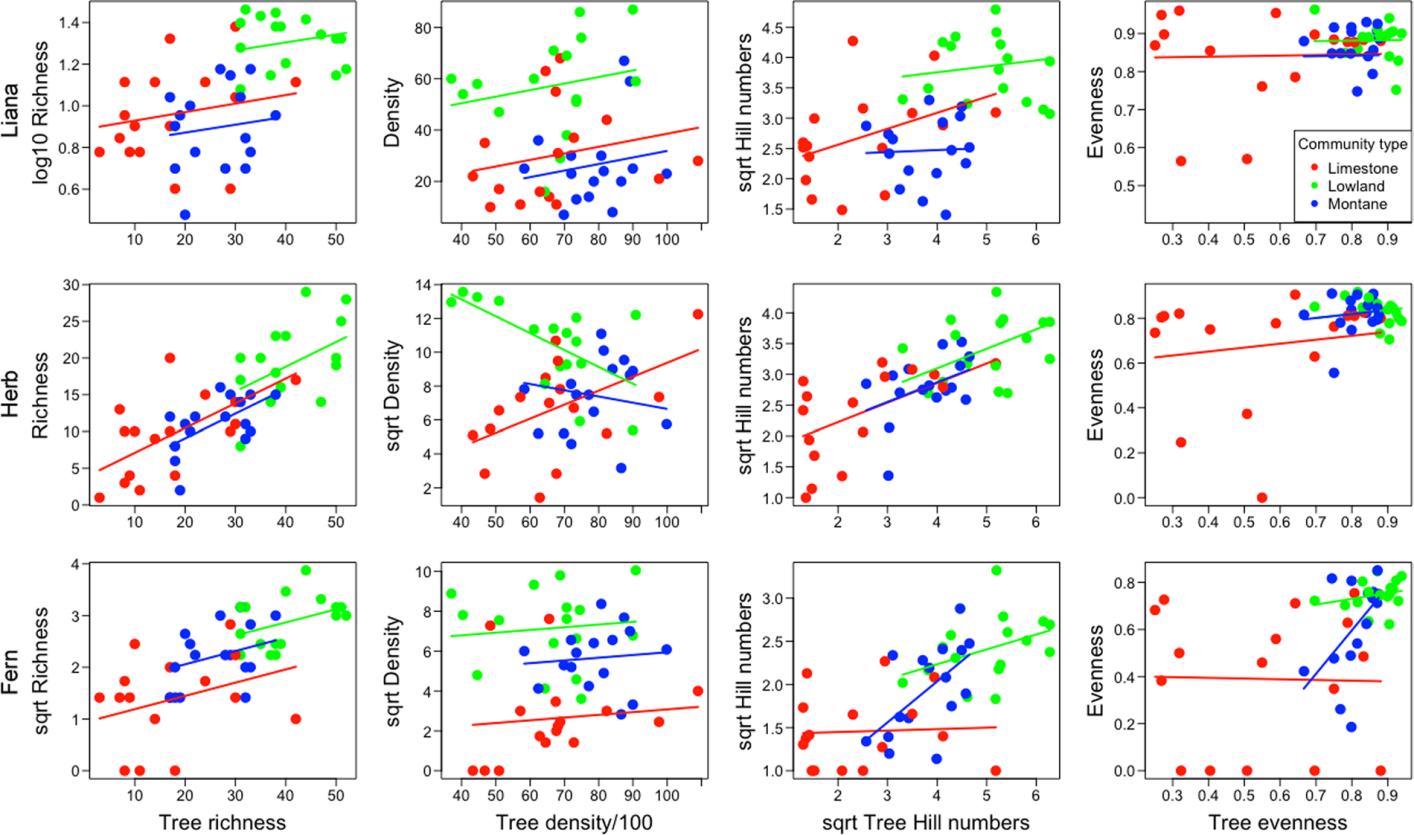
Regression relationships for diversity and density of lianas, herbs, and ferns, modelled against the same indices for trees and grouped by community type (red = limestone forest, green = lowland forest, blue = montane forest). Significant regression lines are shown. Details of chosen statistical models are provided in Table 2 and Supplementary Table S8. Data taken from 48 plots in forest fragments in Xishuangbanna, southwest China.

Next, we checked how well non-tree indices (richness, density, Hill numbers, Pielou’s evenness) could be explained by the equivalent tree indices and the community types. In general, tree indices × community type models explained about half of the data variation, with most R^2^ values ~0.50 (analysis summary in Table 2 and visualisation in Fig. 3; see Supplementary Table S8 for details of coefficient estimates). Prediction power was greatest for life-form richness, in which all models had R^2^ values > 0.50, and lowest for evenness. In terms of component explanatory power, best models for life-form richness were all additive (main effects for community type and tree richness), but most variation was explained by covariation of community type and tree richness. For life-form density, models were additive for lianas and ferns, but interactive for herbs, indicating that the slopes of the herb density–tree density relationships differed by community. In the density models, most explained variation was assigned to community types with a smaller amount explained by tree density. For life-form Hill numbers, interactive models of community type × tree Hill numbers best explained the diversity patterns of lianas and ferns, whereas an additive model was best for herbs. In Pielou’s evenness models, both community type and tree Pielou’s evenness are weak predictors in additive models observed for lianas and herbs, while an interaction model was best for ferns. In Pielou’s evenness models, the variation explained for lianas and herbs (additive models) were both rather low, but high for ferns (interaction model).

**Table 2:** Proportion of sum of squares variation (R^2^) of life-form indices of composition, richness, and density explained by the cognate tree index (Tree), community type (CT) and tree index × community type for best subset models (as indicated in Supplementary Table S8).

Response	Best model	Partitioned variation (R^2^)					Significance (F-test)
		CT	Tree	Covariation (CT, Tree)	CT:Tree	Total	
Richness							
Liana	additive	0.22	0.02	0.27	—	0.51	*p* = <0.001
Herb	additive	0.03	0.19	0.37	—	0.59	*p* = <0.001
Fern	additive	0.09	0.06	0.38	—	0.53	*p* = <0.001
Density							
Liana	additive	0.40	0.03	−0.03	—	0.40	*p* = <0.001
Herb	interactive	0.48	0.01	0.00	0.02	0.51	*p* = <0.001
Fern	additive	0.49	0.01	0.00	—	0.50	*p* = <0.001
Hill numbers							
Liana	interactive	0.26	0.02	0.22	0.03	0.53	*p* = <0.001
Herb	additive	0.01	0.16	0.35	—	0.52	*p* = <0.001
Fern	interactive	0.08	0.05	0.37	0.07	0.56	*p* = <0.001
Pielou’s evenness							
Liana	additive	0.03	0.00	0.01	—	0.04	*NS*
Herb	additive	0.04	0.02	0.13	—	0.19	*p* = 0.020
Fern	interactive	0.15	0.00	0.20	0.06	0.41	*p* = <0.001

Variation explained by main effects is split into exclusive variation for each main effect and shared variation of both main effects (covariation).

### Life-form responses to fragmentation

Finally, to understand whether trees are representative of responses of other life-forms to fragmentation, we tested whether life-forms (trees, lianas, herbs and ferns) were responding to fragmentation in qualitatively similar ways in different communities. We tested this using two fragmentation parameters, associated with each plot, namely fragment size (area) and distance from the plot to the nearest fragment edge, which have both been frequently implicated in community responses to fragmentation^38^.

**Figure 4:**
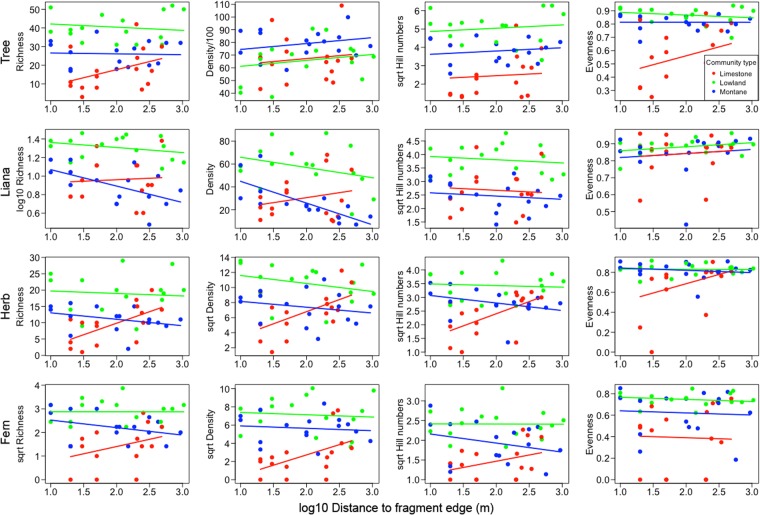
Effects of plot distance to the nearest forest edge on plot life-form diversity and density in different community types (red = limestone forest, green = lowland forest, blue = montane forest). Horizontal lines indicate significant community effects only, parallel and sloped lines indicate significant additive effects of community type and distance to forest edge, and non-parallel lines indicate significant interactions between community type and distance to forest edge. Details of chosen model-averaged statistical models are provided in Table 3 and Supplementary Table S9. Data taken from 48 plots in forest fragments in Xishuangbanna, southwest China.

For all four life-forms, distance to edge had significant effects on diversity indices that often differed by community type (significant interaction), whereas fragment size mostly affected the diversity indices consistently across community types (additive effect) (analysis summary in Table 3 and visualisations in Fig. 4 and Supplementary Fig. S6; see Supplementary Tables S9 and S10 for details of model-averaged coefficient estimates). Within communities, liana responses to distance to fragment edge differed from tree responses for density and evenness, herb responses differed from tree responses for density and Hill numbers, and fern responses differed from tree responses for density, Hill numbers and evenness. With respect to communities, vegetation in limestone forests responded differently to edge distance, with greater density, Hill numbers and evenness further from the edge, whereas patterns in montane and lowland forests followed opposite trends or had very weak trends for all four indices.

**Table 3:** Patterns of composition, richness, and density across the 48 forest plots best explained by models involving community type main effects only (CT), additive models involving community type and fragmentation parameters (FR + CT), or interactive models where responses to fragmentation parameters differed by community type (FR * CT).

	Distance to edge (m)^‡^	**R** ^**2**^	Fragment size (ha)^‡^	**R** ^**2**^
**Richness**				
Tree	FR * CT	0.61	FR + CT	0.55
Liana^‡^	FR * CT	0.56	FR + CT	0.51
Herb	FR * CT	0.51	FR + CT	0.41
Fern^†^	FR * CT	0.53	FR + CT	0.48
**Density**				
Tree	FR + CT	0.17	FR + CT	0.17
Liana	FR * CT	0.50	FR * CT	0.45
Herb^†^	FR * CT	0.44	FR + CT	0.32
Fern^†^	FR * CT	0.56	FR + CT	0.56
**Hill numbers**				
Tree^†^	FR + CT	0.58	FR + CT	0.57
Liana^†^	FR + CT	0.49	FR + CT	0.50
Herb^†^	FR * CT	0.50	FR + CT	0.36
Fern^†^	FR * CT	0.49	FR + CT	0.45
**Pielou’s evenness**				
Tree	FR * CT	0.55	FR + CT	0.51
Liana	FR + CT	0.06	FR + CT	0.09
Herb	FR * CT	0.28	FR + CT	0.19
Fern	FR + CT	0.35	FR + CT	0.36

Best subset models were chosen using ΔAICc comparisons of all subset models (see *Materials and Methods* for further details). All fragmentation parameters tested are shown across the header. ^**†**^Indicates that the variable was square root transformed.

^‡^Indicates that the variable was log10 transformed.

## Discussion

### Community types identified using tree data are representative for the vascular flora

Classifications of the plot data into different plant communities based on non-tree and on tree presence-absence data both identified three forest communities. The major difference between the two analyses was that five plots classified as lowland forest with the non-tree data were classified as montane forest with tree data. These same plots were consistently assigned to lowland forest with liana, herb, and fern data. This shift suggests that these plots lie on the transition between the lowland and montane forest community types, and that trees are more sensitive to increasing elevation. This sensitivity may be because adult trees are exposed to the external climate, whereas the microclimate experienced by herbs, ferns, and some lianas is buffered by the tree canopy. In contrast, the classification differences between limestone and non-limestone communities were very small (one plot moved from limestone forest with non-tree data to lowland forest with tree data), suggesting that soil type drives compositional variation for all life-forms, which were well represented by tree data. Limestone soils are shallow and have low water retention relative to non-limestone soils in Xishuangbanna^39^, so water stress may be a significant driver of community composition on limestone soils.

### Diversity and density patterns of trees poorly represent other plant life-forms

Plot-level tree data was mostly a poor predictor of the diversity or density of other life-forms, based on our regression analyses (Fig. 3 and Table 2). However, the community classifications were similar for tree and all-species data. This shows that the tree indices were indirectly explaining somewhat more of the variation in the data than implied by the direct analysis because of the information accounted for by the community classifications.

Tree richness patterns predicted richness patterns of herbs and ferns quite well, implying that these life-forms were responding similarly to landscape-scale environmental drivers, but they predicted liana richness poorly. Again, since the three identified community types explained up to 49% of the variation in the liana richness, and the community types are fairly well identified by tree data only, tree richness can predict liana richness weakly in the studied landscape.

Tree density predicted the density of other life-forms poorly, in contrast to previous studies^13,40,41^. Again much of the explained variation was due to differences between communities. Previous studies have observed negative relationships between densities of trees and densities of woody lianas^15,42^, but we found weakly positive relationships across community types. One possible explanation for the weak relationship in our data is that large stem density does not predict canopy cover or total biomass, and therefore does not reflect resource changes through the canopy, which in turn determine environmental resource space for lianas. The contrasting relationships between density of trees versus herbs, ferns and lianas across community types (positive for limestone communities and negative for lowland and montane communities) was also surprising. It suggests that understory resource availability within these different communities follows different trends to the resource availability to trees. Further, the fact that this contrasting community pattern was not detected for ferns suggests that ferns and other herbs respond differently to environmental conditions. Both patterns deserve further investigation.

### Trees do not represent the responses of other life-forms to fragmentation

Responses to fragmentation differed by life-form and by plant community type, indicating that tree response to fragmentation was not representative of other life-forms. In general, composition, diversity and density of most life-forms responded weakly to fragment size, with the exception of lianas, which showed strong decreases in density in smaller fragments for montane and lowland forests, but not limestone forests. The absence or weakness of a species-fragment area relationships has also been observed in previous fragmentation studies on plants, and several explanations have been proposed including: delayed responses of plant richness to reduced patch size^26,43^, varied life history strategies of species that result in different responses to changes in fragment size^23,27^, and colonization of light-demanding species and extirpation of shade-adapted species in smaller fragments balancing the number of species dispersed between small and big fragments^24,44^.

Plot distance to fragment edge affected all indices of all life-forms, and differed among community types. Patterns of change were generally stronger in limestone forests than non-limestone forests and often had opposite trends with distance, with indices increasing with increasing distance from edge on limestone and decreasing or not changing with distance on non-limestone. Tree richness patterns were representative of trends for richness among other life-forms, but density, Hill numbers, and Pielou’s evenness of trees were not. Tree density increased moderately with distance from the edge in all forests types, whereas density of other life-forms decreased with distance in montane and lowland forest, but increased rapidly in limestone forest. For tree data, Hill numbers and Pielou’s evenness changed strongly with distance for limestone plots and weakly in the other forest types, but this trend was only repeated for herbs and not for lianas or ferns. This may indicate the shade-tolerant tree and herb species are selected against towards forest edges in limestone fragments and thus become increasingly rare near the edge of the fragments. We expected that trees might be less responsive to the impacts of fragmentation relative to other groups due to their long life spans and slow population turnover^45–47^. The combined information confirms that all life-forms do appear to be responding to edge effects, but the patterns of change differ by life-form. This is somewhat in agreement with the diverse responses to edge effects summarised by Laurence *et al*.^38^.

The strong differences in life-form responses to edge distance in limestone forests versus non-limestone forests, suggest that changes to interior microclimate differ substantially between the parent material types. Limestone karsts tend to be porous and retain soil moisture more poorly than montane or lowland forests^39,48,49^. Thus species may be subject to more severe water restriction at forest edges than interior sites in limestone forest. By contrast, increased canopy openness along forest edges in non-limestone communities, where water availability may be less limiting, may enhance understory plant growth by increasing light availability^28,50^.

### Identifying plant community types is critical for correct inference

In our study system, diversity patterns of plant life-forms and their responses to fragmentation differed strongly among community types. This indicates the importance of first identifying plant communities in landscape-scale forest fragmentation studies and then evaluating changes within those communities separately to fully understand the impacts of forest fragmentation at the landscape scale. This method will also provide additional information about where change is happening in the landscape, facilitating better-targeted and more effective conservation action. For instance, most studies to date have concluded that liana density and richness increase near forest edges and with less forest cover^31,34,51^, but our study shows that this effect is not universal and depends on the community involved. Few research articles on fragmentation at sites other than our own (i.e.^52,53^) have assessed whether there is more than one community type present in the landscape under consideration. One study in the fragmented forest landscape in Chiapas^18^, Mexico, observed that tree diversity responses to forest fragmentation were only detectable within community types and not across the entire landscape. Studies that have been specifically located within a relatively flat, uniform landscape, such as the Biological Dynamics of Forest Fragments Project in central Amazonia^38^, may cover a single community type, but where observations are being made over variable soil types or across elevation (e.g.^54^), we recommend that the plot data first be tested for community types. An unfortunate side effect of accounting for community types in forest fragmentation analyses is that their inclusion severely depletes the statistical power of the analyses, as available replication gets divided among the component communities. This is a severe limitation for community-based studies where replication is always low due to the high time investment required for data collection, and it reduces statistical power to disentangle multiple and likely interacting fragmentation effects (fragment size, edge effects, isolation) directly during inference.

## Conclusion

In this study, we asked whether it is reasonable to rely on tree data to understand patterns of total plant diversity in tropical forests and whether tree data can be used to understand total plant community responses to forest fragmentation. We found that, once community type had been accounted for, tree species richness was a good predictor of herb and fern richness, but a poor predictor of liana richness, and tree density could not predict the density of the other life-forms. Tree responses to fragmentation were also mostly poorly representative of responses of other life-forms to fragmentation, as only tree composition and density were related to fragmentation whereas richness of lianas and herbs were also responding to fragmentation. Thus the effects of fragmentation on forest vegetation cannot be understood through tree responses alone. Moreover, measuring fragmentation at the community level may be critical to detect and understand the effects of fragmentation across forest landscapes, suggesting it is first necessary to identify distinct communities within local landscapes. Communities that are most at risk from fragmentation and other human impacts can be identified using this approach.

## Materials and Methods

### Study area

**Figure 5:**
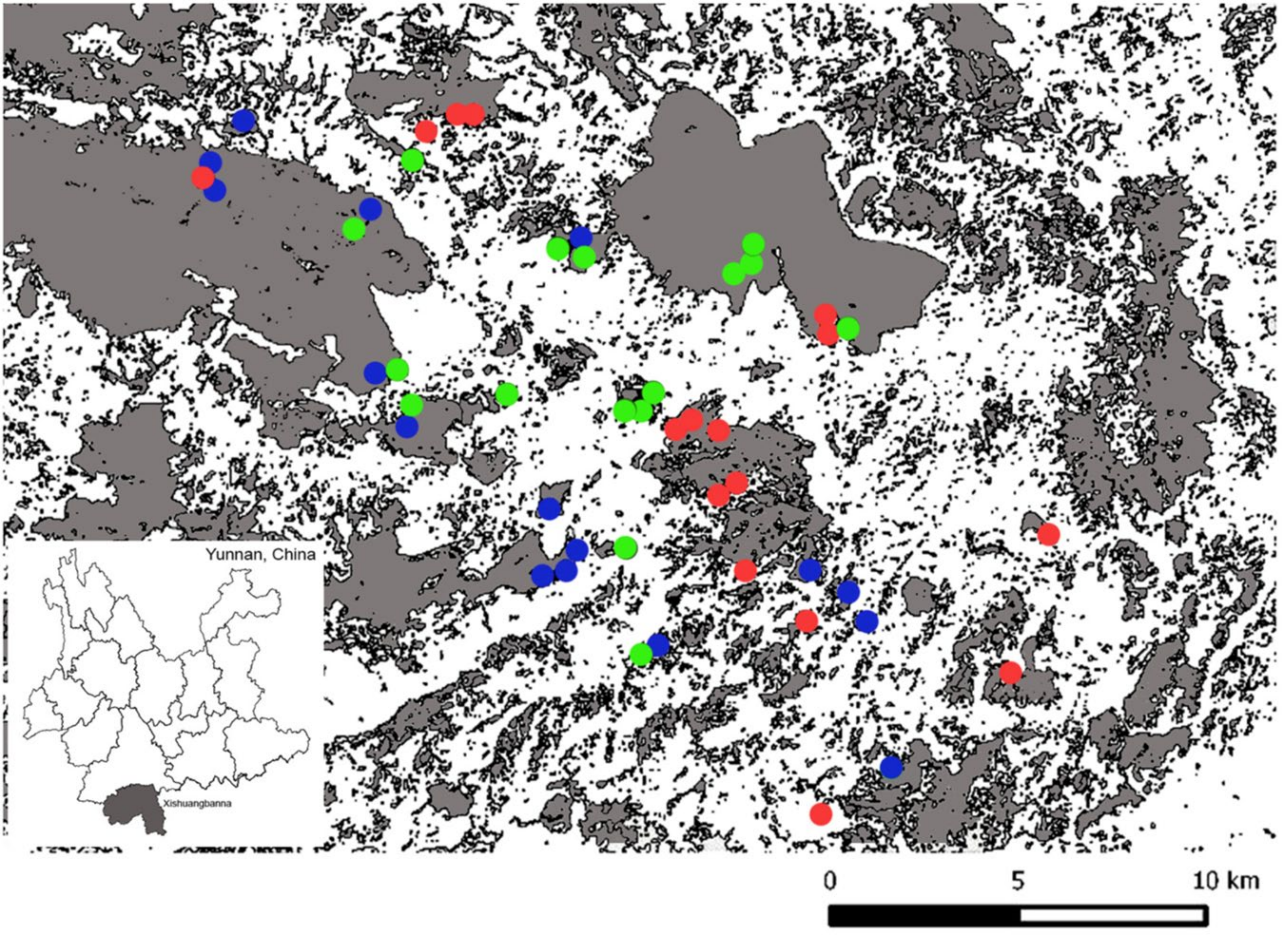
Map showing the location of study area near Menglun Town in Xishuangbanna Dai Autonomous Prefecture, southwest China. Natural forest is shown in grey. The white matrix is predominantly made up of rubber plantation (*Hevea brasiliensis*) and urban settlements. Forty-eight (48) plots are located in twenty-three (23) forest fragments. The larger fragments contain multiple plots as these are located across topographical elements in the landscape. The different types of forest identified at each plot by hierarchical clustering on principal components (HCPC – see Methods) are indicated using separate colours: red = limestone forest (16 plots); green = lowland forest (16 plots); blue = montane forest (16 plots).

In 2012 we established 50 permanent study plots in 23 forest fragments within a 10-km radius around Menglun Town (21°54′57″ N, 101°16′12″ E) in Xishuangbanna Dai Autonomous Prefecture, Yunnan, Southwest China (see ref.^53^ for detailed study plot description) (Fig. 5). The region has mountainous topography and a seasonal climate, with a hot wet season from May to October and a cooler dry season from November to April. The mean annual rainfall is 1454 mm, of which 87% falls in the rainy season (May to October)^55^. We deliberately sampled plots at different elevations and topographic positions across and within fragments to capture perceived variation in the landscape. These plots cover an elevation range of 541–1477 m, across limestone karst and sandstone substrates^48^, and are located in a range of patch sizes (0.09 to 13873 ha). Subsequent to 2012, two of the forest fragments were destroyed, leaving 48 plots for our analysis (the data was collected in 2013 and 2014).

The tropical forests of Xishuangbanna have the highest plant diversity in China^56^, but forest cover in Xishuangbanna has declined from ~70% to 40% over the last 30 years due largely to the expansion of monoculture rubber farming^57^. Nature Reserves protect 15.5% of Xishuangbanna’s land area, including the largest remaining forest areas, but numerous unprotected forest fragments of various sizes persist in the agricultural matrix. In our study area the predominant land-use is rubber (*Hevea brasiliensis*) plantation, which abuts most forest edges near our sampling plots. The forest areas converted to rubber plantations in Xishuangbanna were largely in the most agriculturally favourable locations, leaving these remaining forest fragments on steeper slopes, poorer soils, and shaded aspects^53^.

The 50 plots were distributed over the landscape with the aim of covering a range of patch sizes (0.09 to 13873 ha), elevations (541–1477 m a.s.l.), topography, and aspects. Within fragments, plots were established in visually intact forest cover, 10–1047 m from forest edges. The bigger forest fragments (>100 ha) are designated forest reserves, whereas most of the smaller fragments were unprotected: in 2013, two of these plots were destroyed when the forest fragments they were located in were cut down for plantations, thus leaving 48 plots for the analysis (see Supplementary Table S4, for site characteristics of the plots).

### Vegetation Sampling

**Figure 6:**
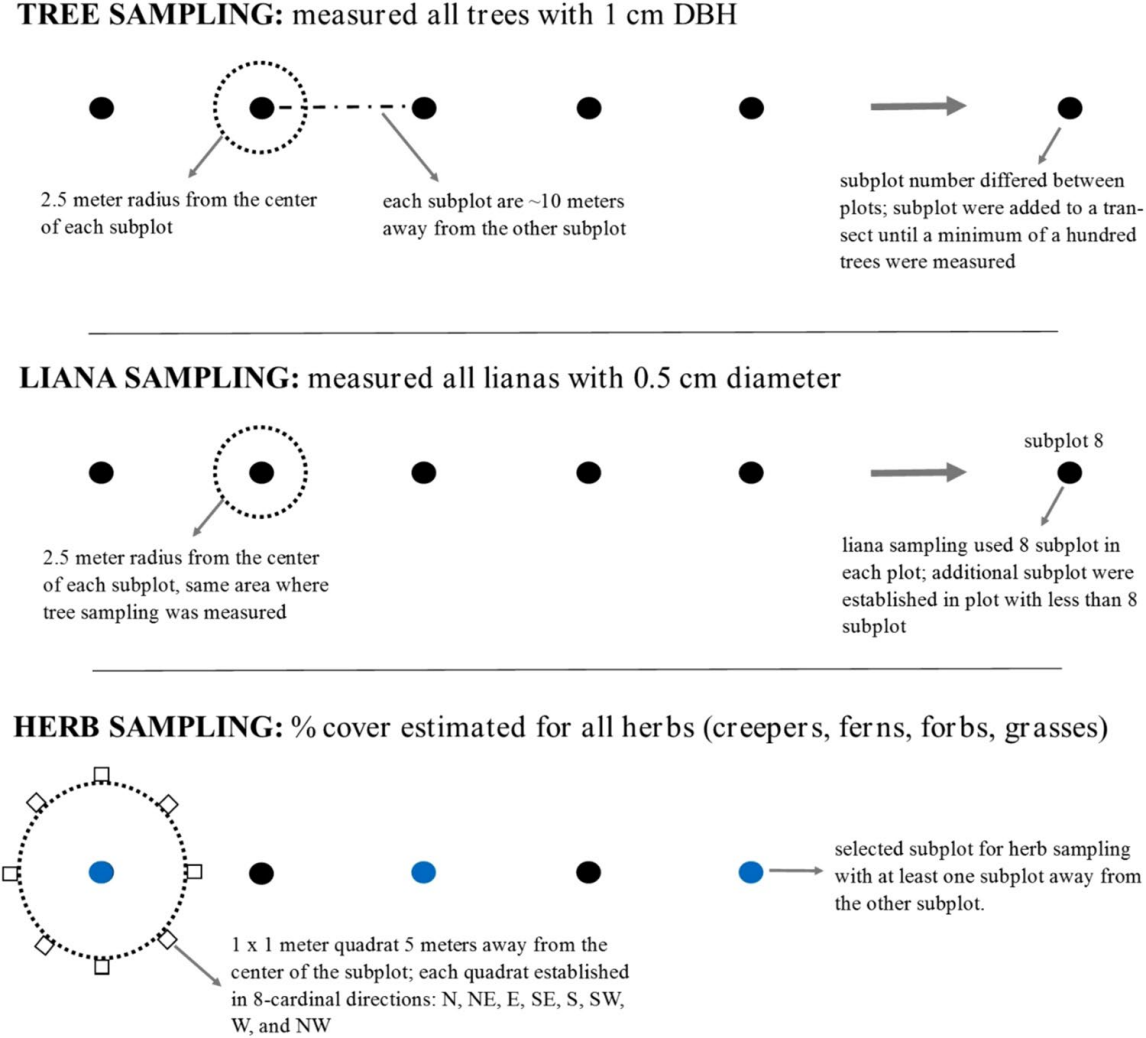
Different sampling protocols for trees, lianas, and herbs in the established fragment plots in Xishuangbanna, Southwest China.

Data for trees, herbs and lianas was collected using different protocols (Fig. 6). We initially established the forest fragment plots in 2012 to study tree diversity^53^. We sampled the tree diversity using a variable area approach where 2.5-m radius circular subplots were added at 10-m intervals to a transect until a minimum of 100 trees (≥1 cm DBH) were tagged and measured, yielding 5–15 circular subplots in each plot. Sub-plots were set in a straight line, which should have limited bias in sampling between larger and smaller trees. We recorded the GPS location for each plot at the centre of the subplot transect. We have re-measured the trees annually, but we use the 2013 data for this study for comparability with the data for other life-forms.

In 2013, we recorded herb and fern composition and diversity in each plot by selecting three circular subplots from the subplots previously used for tree sampling (the two end sub-plots and the central sub-plot). Each selected subplot was at least one subplot away from the other two selected subplots. To avoid disturbing the 2.5 m radius tree sampling subplot, we established eight 1 × 1 m quadrats 5 m from the subplot centre in eight cardinal directions (i.e. N, NE, E, SE, S, SW, W, NW). Within each quadrat, we estimated percentage cover of all vascular herbs of the forest floor, including creepers (lianas that were growing prostrate on the ground surface), grasses, forbs, and ferns, that had predominantly green, non-woody stems. Herbaceous lianas represented 18.7% of the herb data. We sampled the herbs and ferns twice in the year, during the late dry season (March–May) and late wet season (September–October), to detect as many species as possible (some herb species are deciduous and therefore not detectable all year).

We surveyed woody lianas from January to April 2014 in the plots, using eight consecutive 2.5-m radius subplots in each plot. We used the same subplots as for the tree survey, but established new subplots if there were fewer than eight, so that a total of 157 m^2^ was sampled in each plot. All lianas ≥ 0.5 cm diameter were included, following the census protocols of Gerwing, *et al*.^58^ and Schnitzer, *et al*.^59^.

We collected specimens of all morphospecies identified in each plot. Subsequently we carefully matched all species and morphospecies across plots. We identified the matched plant specimens through consultation with experts, comparison with herbarium specimens at Xishuangbanna Tropical Botanical Garden (HITBC), and on-line, using the keys and descriptions in the recently completed Flora of China (http://www.efloras.org/). For some of the lianas we could only find stem material and no leaves, rendering matching on morphology impossible, so we collected DNA samples from those stems and used sequenced *rbcL* barcodes to identify species against Genbank records. All plant specimens are stored in the Community Ecology and Conservation Laboratory, Xishuangbanna Tropical Botanical Garden, Chinese Academy of Sciences, Yunnan, China.

We recorded 789 species in our 48 forest fragment plots, including 421 trees, 163 lianas, 139 herbs, and 66 ferns.

### Life-form diversity and density measures

We calculated several indices that describe the diversity of the life-forms in each plot (data in Supplementary Table S5). Richness was the number of species we recorded in each fragment plot. From the composition data, we calculated Shannon diversity indices, Pielou’s evenness indices, Fisher’s alphas, and Hill numbers for each life-form in each plot.

We checked the collected plot data for completeness in two ways. We constructed species accumulation curves for each of the life-forms (Fig. S4) and we also estimated a number of different richness estimators that test for sampling completeness (including Chao1 and Chao2, as appropriate to the way the data was sampled) (Fig. S5). Species accumulation curves flattened off for lianas, herbs and ferns but not for trees. This is unsurprising as total tree richness we measured (421 morphospecies) was greater than combined total richness of lianas, herbs and ferns (368 species). However tree counts were fixed to accumulate at least 100 stems, so richness found with the tree data is equivalent to what would be observed with rarefaction, thus the observed diversity values for trees are still meaningful and comparable. Because we sampled trees over different numbers of subplots (to reach at least 100 stems) there was a possibility that this caused bias in the estimates. We checked for bias by regressing plot diversity measures for tree data against sub-plot number per plot (Supplementary Table S6). No measures were significantly correlated with the number of subplots, thus no bias was apparent.

Next, we checked correlations between all diversity indices in each life-form (Supplementary Table S7). Richness, Shannon diversity, Fisher’s alpha and Hill numbers were all highly correlated with one another for all life-forms. Hill numbers are thought to be a more reliable estimate of effective diversity^60,61^. Therefore we only used Hill numbers and Pielou’s evenness in subsequent analyses.

Life-form density in each plot were calculated using species abundance data for each plot and were defined differently according to life-form as they were sampled in different ways. Tree densities were adjusted to stems per hectare, since trees were sampled for a fixed number of stems but different numbers of circular plots (stem number/plot surface area * 10,000). Liana density was the total number of stems in the eight circular sub-plots as the total area sampled for lianas was fixed per plot (stem number per 157 m^2^). Herb and fern abundance were not based on stem counts, since many were difficult to count and some had no stems, but were based on their frequency of occurrence (presence-absence) within the forty-eight 1 × 1 m quadrats in each plot (48 m^2^ per plot).

### Statistical analyses

We conducted all analyses in the statistical program R^62^, including the *vegan* package for diversity calculations^63^ and the *FactoMineR* package for clustering^36^, and the basic statistical utilities in R for linear model analysis.

#### Representativeness of trees for community classification

We identified community types in our study area by using hierarchical clustering with principal components (HCPC), using the *FactoMineR* package^36^, to cluster the plots into groups of similar composition. The HCPC method first clusters the data in a hierarchical tree using Ward’s criterion, then the identified clusters are mapped onto the PCA axes generated by the same data and K-means clustering re-assigns individual plots to different clusters based on the nearest cluster means to each plot. We used presence-absence data rather than abundance data for the cluster analysis. Much has been written about the appropriateness of using presence-absence versus abundance data (e.g.^64^). Our intent here was to understand community identity based on species membership rather than on dominance, which might itself reflect responses to finer-scale environmental gradients and fragmentation parameters. The abundance data itself is used in density, Hill number and Pielou’s evenness, so is conveyed in subsequent analyses.

Our first question pertained to whether community types generated by tree data were representative of community types generated by the other life-forms. Therefore, we generated clusters via HCPC for 6 data sets: tree data only, liana data only, herb data only, fern data only, and all life-forms except trees data (“non-tree” data). Additionally, we specified the number of clusters to be generated in each data set to range from 2 to 8 (thus calculating 7 clustering results for each data set). Then, to measure how well clusters generated by the tree data represented equal numbers of clusters generated by each of the other four data sets, we assessed the ability of the tree data to recover the clusters generated by the other groups using confusion matrices generated for each specified number of clusters. Confusion matrices (also known as error matrices) assess the ability of a data set to predict ‘actual’ values. For our problem we treated the clusters generated by tree data as the predictor data and the clusters generated by each of the other life-forms as the ‘actual’ data. The confusion matrix measures how well the predicted class performs in assigning each data point (our plots) to the correct class as specified by the ‘actual’ data. Using the confusion matrix, we calculated the precision (=true positives/(true positives + false positives), sensitivity (the true positive rate, = true positives/(predicted positives)), and F1 (the harmonic mean of precision and sensitivity) measures based on those confusion matrices^65^. We graphed the confusion matrix measures for each tree – life-form comparison.

#### Environmental drivers of the community types

We selected the community types based on the clusters generated by HCPC for the all life-forms data, as this was the most extensive species list. We anticipated that forest diversity in Xishuangbanna was structured both by elevational changes, topographical location (valley, slope or ridge) and limestone versus non-limestone soils (measured in our plots as pH) (data presented in Table S4). Therefore, we tested whether the clusters generated by the HCPC could be differentiated with respect to the three environmental variables. We regressed the ordinal variables, elevation and pH, each as a function of the generated clusters and performed pairwise comparisons using Tukey’s HSD tests. We fit a multinomial model between the topographic elements and the assigned groups, using the *multinom*() function in the *nnet* package^66^, to generate a probability for the distribution of each community type in relation to each topographic element.

#### Representativeness of trees for plant life-form diversity and density

To assess whether patterns of tree richness, density, and species composition in different plant community types could predict the same indices in lianas, herbs and ferns, we modelled each index (richness, Hill numbers, Pielou’s evenness, and density) for each life-form group (LF_I_) against the cognate index for trees (T_I_) and the identified community types (CT) in a full interaction model (eq. 1).

1 $$L{F}_{I} \sim {T}_{I}+CT+{T}_{I}:CT+\varepsilon $$

We selected informative predictors in the maximal model using an information theoretic approach^67^. Details of the method are provided further down the text.

#### Response of life-forms to fragmentation among community types

To assess whether life-forms (trees, lianas, herbs and ferns) were responding to fragmentation in a similar way, we modelled each index for each life-form group against each fragmentation effect (FR) and the community types in a full interaction model (eq. 2):

2 $$L{F}_{I} \sim FR+CT+FR:CT+\varepsilon $$

We ran these models separately for each of five fragmentation predictors: patch size (m^2^); distance from the centre of the plot to the nearest fragment edge (m). Patch size and distance to edge were measured using Fragstat 4.0^68,69^.

Prior to model evaluation, we checked whether any of the fragmentation parameters were correlated with plot elevation or soil pH; none were (all rho values <0.28, all t-tests NS). We also checked whether fragmentation parameters differed with respect to plot topographic location using ANOVA; none of the fragmentation parameters differed significantly with respect to topography (F-values all NS). We selected informative predictors in the model using an information theoretic approach^67^. Details of the method are provided next.

#### Selection of informative predictors and estimated effects from maximal models

Before proceeding to model selection we first checked whether autocorrelation and pseudoreplication in the data explained sufficient amounts of variation in the data to warrant accounting for them in the model analyses.

First, because our plots were clustered in the landscape (see Fig. 5), we checked whether autocorrelation in the data explained significant amounts of variation. We ran generalised least squares models (using the gls() function in the R package *ape*^70^), using the longitude and latitude of the plots to construct correlation matrices and hence all possible correlation structures offered with the function, as well as a model with the identity matrix (no correlation). We then compared the models using the generated AIC per model. We did this for all possible richness and density responses of all life-forms. In all cases the model with identity correlation had the lowest AIC, leading us to conclude that there was no need to explicitly account for spatial autocorrelation in model structures. So we did not proceed with model autocorrelation.

Second, because multiple plots were selected from the same fragments (see Fig. 5), we assessed whether it was necessary to fit a mixed effects model to account for the non-independence in the data due to within-plot replication,. We ran these tests using the full model where we included fragment identity as a random effect. We ran these models using the lmer() function of the package *lme4*^71^. We assessed whether the random effect was explaining significant variation in the data by running the confint() function using parametric bootstrapping with 5000 simulations, and checked whether the 95% confidence interval the random effect variance was distinct from zero. We did this for all possible richness and density responses of all life-forms. In all cases the confidence interval of the random effect variance included zero, suggesting that the random effect did not explain significant variation in the data. Therefore, we proceeded with model selection using the linear models (lm() function in the base packages of R).

Third, after confirming that pseudoreplication and autocorrelation were not explaining significant amounts of the data variation, we proceeded to choose informative predictors using an information theory approach^67^ conducted with the ‘*MuMIn’* package^72^. The method gives all possible subset model combinations of the predictors in the maximal models (equations 1 and 2) and grades the models according to the adjusted Akaike Information Criterion used for smaller sample sizes (AICc)^73^, and weights the models according to the difference between each model’s AICc and the AICc of the best model (ΔAICc). Then we used model averaging based on the Akaike weights to generate weighted coefficients and standard errors for the predictors found in models with ΔAICc < 4^67^. We also report the importance value of each predictor retained in this final predictor set. To evaluate how well the predictors could potentially explain the response data, we recorded the R^2^ value of the maximal model (as defined in Equations 1 and 2 above). We graphed the chosen model-averaged coefficients of the selected predictors against the data to visualise the relationships.

### Data availability

Individual plot life-form diversity and density data, and environmental data are provided in the Supplementary Information of this article. Individual plot composition data may be requested from the corresponding author (kyle.tomlinson@xtbg.org.cn).

## Electronic supplementary material

Supplementary Information
