# Supplementary material for: Trees represent community composition of other plant life-forms, but not their diversity, abundance or responses to fragmentation
Source: Sci Rep. 2018 Jul 27;8:11374. doi: 10.1038/s41598-018-29635-9 (PMC6063943; doi:10.1038/s41598-018-29635-9)
Supplement: Supplementary file 1 — Supplementary Information [file 41598_2018_29635_MOESM1_ESM.docx]

**Supplementary Information**

**Trees represent community composition of other plant life-forms, but not their diversity, abundance or responses to fragmentation**

Bonifacio O. Pasion^1,2^, Mareike Roeder^1^, Jiajia Liu^3^, Mika Yasuda^4^, Richard T. Corlett^1^, J.W. Ferry Slik^5^ and Kyle W. Tomlinson^1,*^

^1^Center for Integrative Conservation, Xishuangbanna Tropical Botanical Garden, Chinese Academy of Sciences, Menglun, Mengla, Yunnan 666303, China

^2^University of the Chinese Academy of Sciences, Beijing, China

^3^Key Laboratory of Conservation Biology for Endangered Wildlife of the Ministry of Education, College of Life Sciences, Zhejiang University, Hangzhou, China

^4^Birdlife International Tokyo, 4F TM Suidobashi Bldg., 2-14-6 Misaki-cho, Chiyoda-ku, Tokyo 101-0061, Japan

^5^Faculty of Science, Environmental and Life Sciences, Universiti Brunei Darussalam, Brunei Darussalam


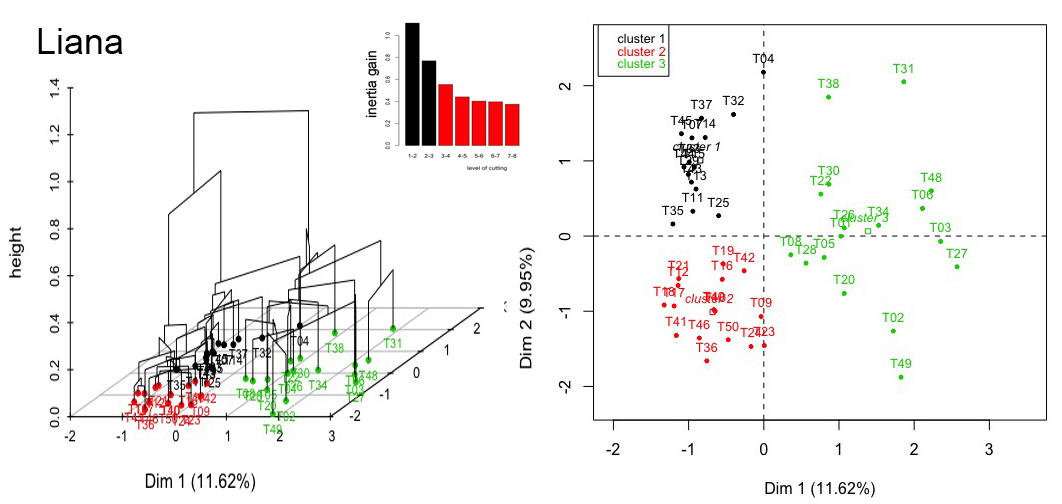


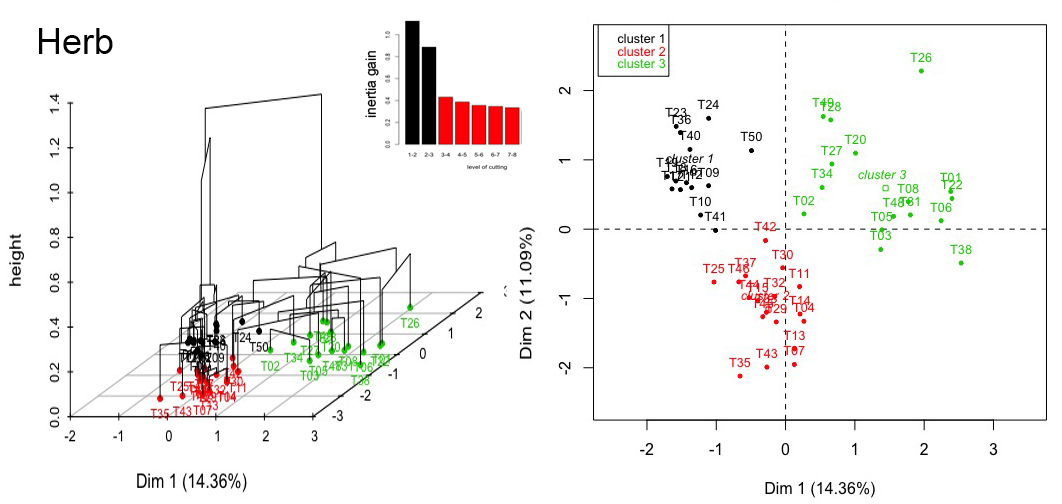


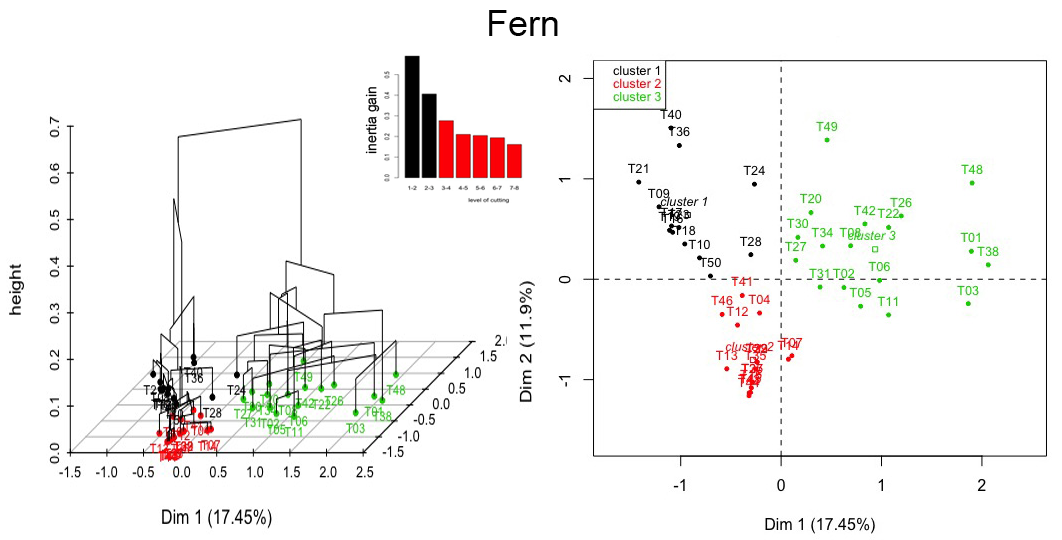


**Figure S1 a-c.** Clusters generated by HCPC on the presence-absence data for lianas (a), herbs (b), and ferns (c). Clusters are first selected using hierarchical clustering with Ward’s criterion and specified number of clusters. Subsequently individual sites may be reassigned to other groups on the basis of K-means clustering. All figures show clustering with three groups specified. Inertia graphics generated for each life-form indicate that inertia is best accounted for by four groups for all life forms.

**
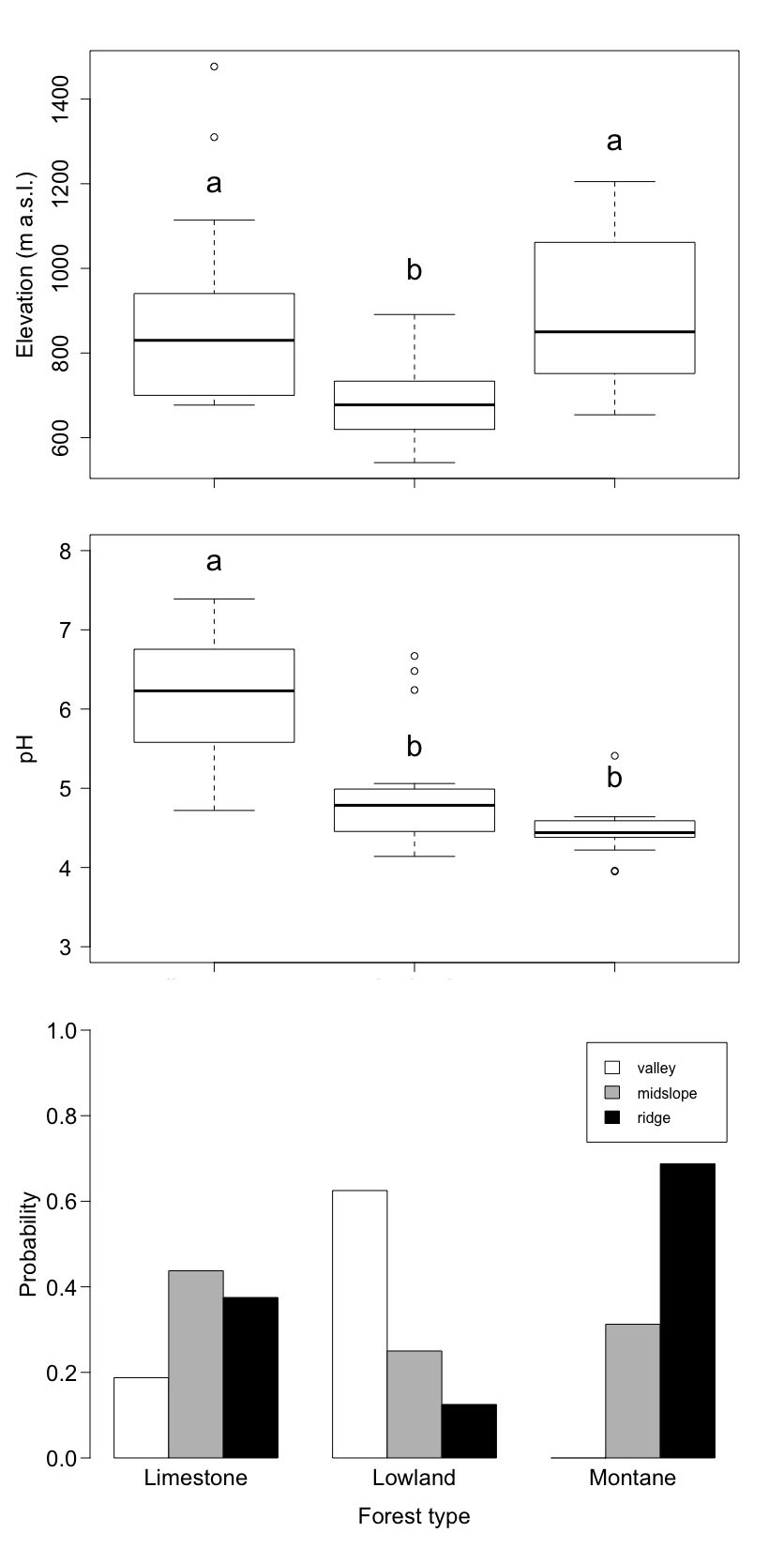
**

**Figure S2.** Environmental variables that differ between identified community types. a. pH. b. elevation (mean altitude above sea level). c. Topographic location. Letters above box plots for pH and elevation indicate significantly different groups based on Tukey HSD pairwise tests of means. Probabilities of occurrence of each community type at each topographic location in the landscape are based on multinomial analysis[^67^](#_ENREF_67).

**
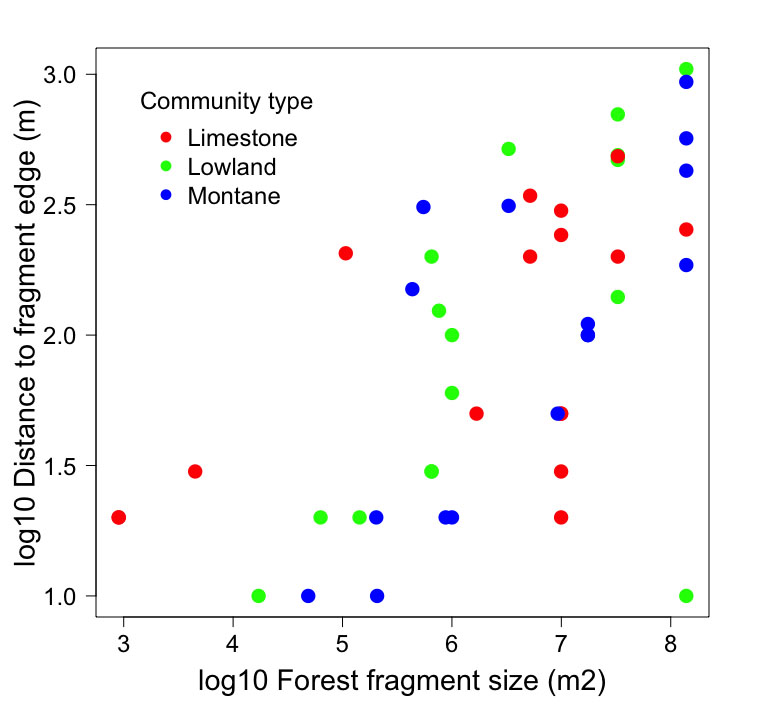
**

**Figure S3.** Distribution of plots of different forest community types across forest fragment sizes and across distance to edge. The forest communities did not differ with respect to mean forest fragment size (F_2,44_ = 0.497, P = 0.612) or mean distance from plot to fragment edge (F_2,44_ = 0.025, P = 0.975).


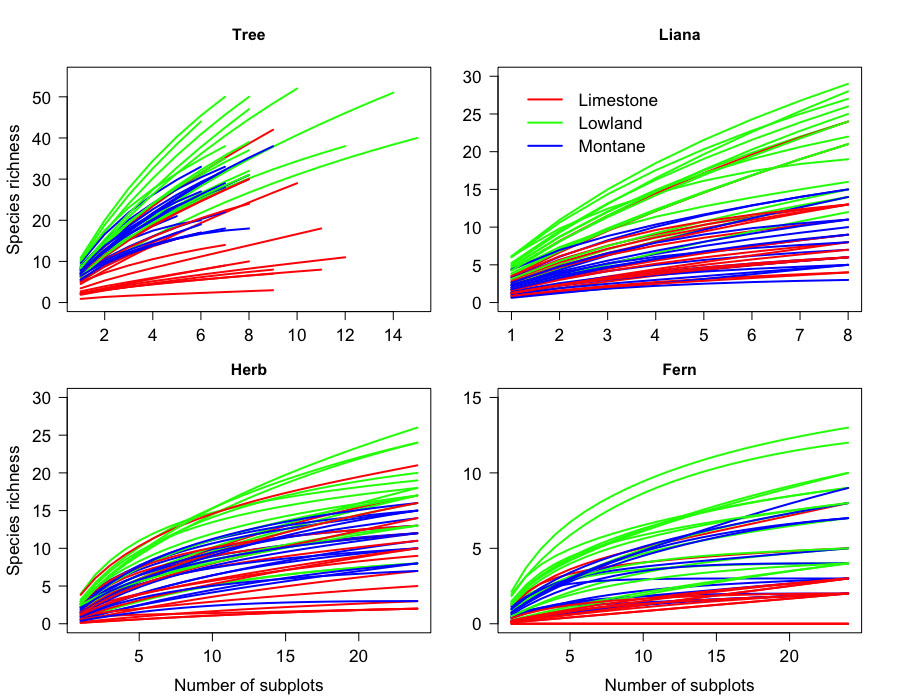


**Figure S4.** Species accumulation curves for each life-form in each sampling plot. Colours indicate the assigned community type.


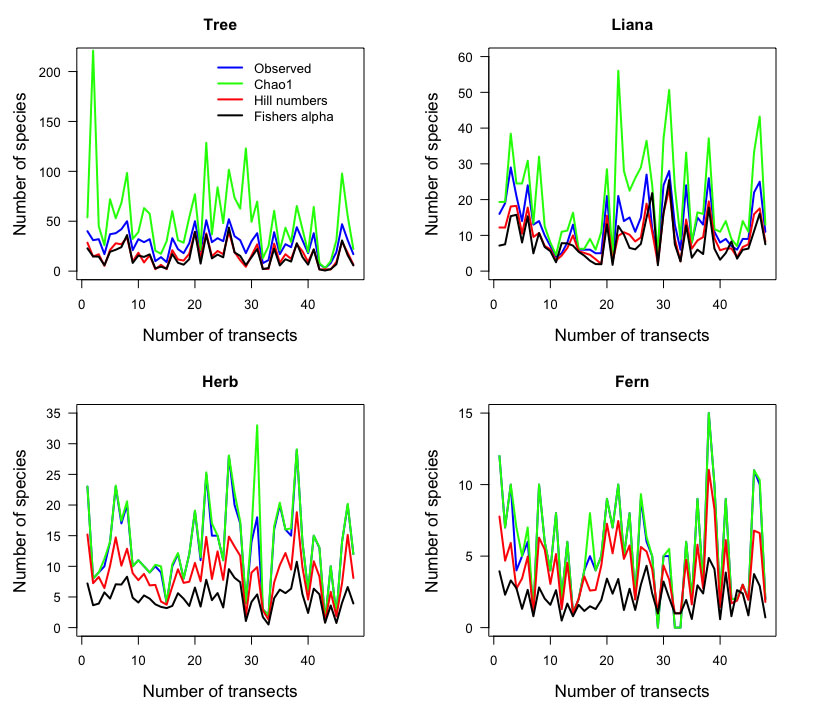


**Figure S5.** Observed richness and predicted richness based on standard richness measures. The Chao1 results suggest that total community richness is undersampled for trees but not for lianas, herbs and ferns. Hill expected richness and Fisher’s alpha follow trends that are similar to the observed richness.

**
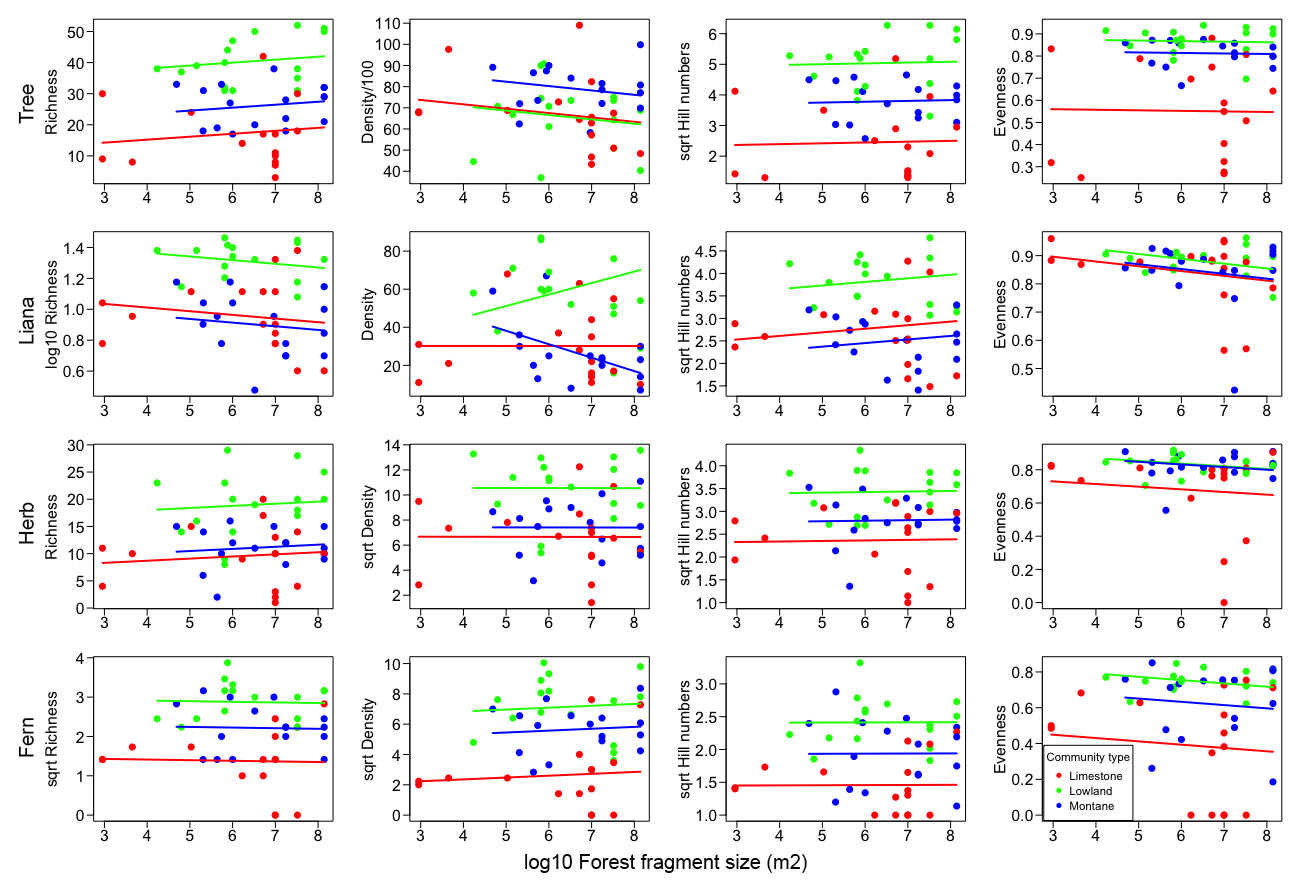
**

**Figure S6.** Effects of fragment size on plot life-form diversity and density in different community types (red = limestone forest, green = lowland forest, blue = montane forest). Horizontal lines indicate significant community effects only, parallel and sloped lines indicate significant additive effects of community type and fragment size, and non-parallel lines indicate significant interactions between community type and fragment size. Details of chosen model-averaged statistical models are in Table 2 and Supplementary Table S10. Data taken from 48 plots in forest fragments in Xishuangbanna, southwest China.

**Table S1.** Evaluation of ability of tree species data to recover the community clusters, identified by the other life-forms based on presence-absence matrices (All = all species data including trees, Non-tree = all species data except tree data), using confusion matrix measures. Each comparison was run multiple times with the number of clusters fixed from 2 to 8 clusters. Confusion matrix measures used were: P = Precision (the fraction of correct predictions for a certain class), R = sensitivity (recall) (the fraction of instances of a class that were correctly predicted, FP = False positive, F1 = F1-score (the harmonic mean of precision and recall)^66^. All measure values are proportional.

| Lifeform | Evaluation Index | Number of clusters | | | | | | |
| --- | --- | --- | --- | --- | --- | --- | --- | --- |
|  |  | 2 | 3 | 4 | 5 | 6 | 7 | 8 |
| All  vs tree | P | 0.44 | 0.89 | 0.97 | 0.75 | 0.73 | 0.64 | 0.64 |
|  | R | 0.44 | 0.88 | 0.94 | 0.88 | 0.67 | 0.70 | 0.70 |
|  | FP | 0.66 | 0.06 | 0.01 | 0.02 | 0.02 | 0.03 | 0.03 |
|  | F1 | 0.43 | 0.87 | 0.95 | 0.89 | 0.86 | 0.84 | 0.84 |
|  |  |  |  |  |  |  |  |  |
| Non-tree vs tree | P | 0.86 | 0.89 | 0.71 | 0.64 | 0.69 | 0.62 | 0.56 |
|  | R | 0.91 | 0.88 | 0.63 | 0.77 | 0.58 | 0.59 | 0.56 |
|  | FP | 0.19 | 0.06 | 0.06 | 0.03 | 0.03 | 0.03 | 0.03 |
|  | F1 | 0.87 | 0.87 | 0.88 | 0.78 | 0.75 | 0.72 | 0.73 |
|  |  |  |  |  |  |  |  |  |
| Liana vs tree | P | 0.42 | 0.87 | 0.76 | 0.61 | 0.52 | 0.52 | 0.45 |
|  | R | 0.42 | 0.86 | 0.75 | 0.75 | 0.55 | 0.57 | 0.48 |
|  | FP | 0.65 | 0.07 | 0.06 | 0.04 | 0.04 | 0.03 | 0.03 |
|  | F1 | 0.42 | 0.85 | 0.73 | 0.74 | 0.71 | 0.75 | 0.74 |
|  |  |  |  |  |  |  |  |  |
| Herb vs tree | P | 0.42 | 0.86 | 0.82 | 0.63 | 0.56 | 0.50 | 0.60 |
|  | R | 0.41 | 0.84 | 0.85 | 0.71 | 0.77 | 0.49 | 0.51 |
|  | FP | 0.41 | 0.83 | 0.78 | 0.71 | 0.73 | 0.71 | 0.58 |
|  | F1 | 0.72 | 0.09 | 0.06 | 0.05 | 0.05 | 0.04 | 0.04 |
|  |  |  |  |  |  |  |  |  |
| Fern  vs tree | P | 0.41 | 0.81 | 0.73 | 0.62 | 0.75 | 0.66 | 0.38 |
|  | R | 0.40 | 0.79 | 0.72 | 0.72 | 0.80 | 0.63 | 0.47 |
|  | FP | 0.64 | 0.12 | 0.08 | 0.06 | 0.05 | 0.04 | 0.04 |
|  | F1 | 0.40 | 0.77 | 0.71 | 0.67 | 0.76 | 0.72 | 0.60 |

**Table S2.** Richness and density for each plant life-form (tree, liana, herb and fern) in three community types: limestone forest, lowland forest, and montane forest. Values are estimated means with standard errors (mean + SE). Different letters (a-c) indicate significant differences between community types as assessed with Tukey’s HSD tests.

|  | Limestone | Lowland | Montane |
| --- | --- | --- | --- |
|  | n=16 | n=16 | n=16 |
| *Richness* |  |  |  |
| Tree | 17.3 + 2.7^a^ | 40.4 + 1.9^b^ | 26.1 + 1.7^c^ |
| Liana | 10.4 + 1.4^a^ | 21.1 + 1.4^b^ | 8.7 + 0.9^a^ |
| Herb | 9.6 + 1.4^a^ | 18.9 + 1.5^b^ | 11.1 + 0.9^a^ |
| Fern | 2.6 + 0.6^a^ | 8.5 + 0.7^b^ | 5.2 + 0.7^c^ |
|  |  |  |  |
| *Density* |  |  |  |
| Tree | 6707.1 + 441.3^a,b^ | 6577.6 + 393.5^a^ | 7893.0 + 269.3^b^ |
| Liana | 30.2 + 4.7^a^ | 57.1 + 4.8^b^ | 26.5 + 4.1^a^ |
| Herb | 52.1 + 9.9^a^ | 117.2 + 12.2^b^ | 59.4 + 7.9^a^ |
| Fern | 11.6 + 4.4^a^ | 54.8 + 6.9^b^ | 34.1 + 4.2^c^ |
|  |  |  |  |
| *Hill numbers* |  |  |  |
| Tree | 7.39 + 1.81^a^ | 26.15 + 2.19^b^ | 14.80 + 1.21^c^ |
| Liana | 7.75 + 1.12^a^ | 14.77 + 1.02^b^ | 6.37 + 0.66^a^ |
| Herb | 6.12 + 0.80^a^ | 12.00 + 0.87^b^ | 8.11 + 0.66^a^ |
| Fern | 2.29 + 0.34^a^ | 5.96 + 0.48^b^ | 4.01 + 0.51^c^ |
|  |  |  |  |
| *Pielou's evenness* |  |  |  |
| Tree | 0.56 + 0.06^a^ | 0.87 + 0.02^b^ | 0.81 + 0.01^b^ |
| Liana | 0.84 + 0.03^a^ | 0.88 + 0.01^a^ | 0.84 + 0.03^a^ |
| Herb | 0.68 + 0.06^a^ | 0.83 + 0.01^b^ | 0.82 + 0.02^b^ |
| Fern | 0.39 + 0.07^a^ | 0.75 + 0.02^b^ | 0.62 + 0.05^b^ |

**Table S3**. Representative species of the three plant community types in the study area in Xishuangbanna, Yunnan, China. Listed species are at least 5% of the total stems (for trees and lianas) and of total frequency (for herb and fern) in each plot.

| Life form | Limestone | Lowland | Montane |
| --- | --- | --- | --- |
| Tree | *Cleistanthus sumatranus* (44%) | *Millettia leptobotrya* (8%) | *Castanopsis fleuryi* (13%) |
|  | *Lasiococca comberi* (9%) | *Pittosporopsis kerrii* (6%) | *Millettia leptobotrya* (10%) |
|  | *Celtis philippensis* (6%) |  | *Aporosa yunnanensis* (9%) |
|  |  |  | *Castanopsis echinocarpa* (8%) |
|  |  |  |  |
| Liana | *Quisqualis cf caudata* (10%) | *Piper flaviflorum* (11%) | *Smilax hypoglauca* (10%) |
|  | *Loeseneriella yunnanensis* (9%) | *Benkara sinensis* (8%) | *Smilax glabra* (9%) |
|  | *Derris caudatilimba* (6%) |  | *Dalbergia stipulacea* (8%) |
|  | *Amalocalyx microlobus* (6%) |  | *Craspedolobium unijugum* (7%) |
|  | *Byttneria aspera*  (6%) |  | *Celastrus monospermus* (5%) |
|  |  |  |  |
| Herb | *Pseuderanthemum polyanthum* (26%) | *Piper sarmentosum* (14%) | *Scleria ciliaris* (14%) |
|  | *Oplismus compositus* (8%) | *Amomum villosum* (12%) | *Thysanolaena latifolia* (9%) |
|  | *Psychotria prainii* (5%) | *Pseuderanthemum polyanthum* (10%) | *Cyrtococcum patens* (9%) |
|  |  | *Stachyphrynium placenarium* (9%) | *Alpinia conchigera* (9%) |
|  |  |  | *Smilax lanceifolia* (8%) |
|  |  |  | *Microstegium ciliatum* (7%) |
|  |  |  |  |
| Fern | *Adiantum soboliferum* (31%) | *Bolbitis heteroclita* (18%) | *Lygodium flexuosum* (28%) |
|  | *Selaginella pulvinata* (26%) | *Tectaria herpetocaulos* (10%) | *Lindsaea ensifolia* (23%) |
|  | *Adiantum malesianum* (11%) | *Lygodium flexuosum* (9%) | *Woodwardia japonica* (10%) |
|  | *Lygodium flexuosum* (11%) | *Selaginella delicatula* (7%) | *Adiantum flabellulatum* (6%) |
|  | *Asplenium saxicola* (8%) | *Christella arida* (7%) | *Dicranopteris ampla* (6%) |
|  |  | *Pteridrys australis* (7%) | *Lygodium polystachyum* (5%) |

**Table S4.** Description of the 48 plots based from plant community classification and environmental parameters (Edge = plot distance to fragment edge; Fragment = forest fragment in which plot is located).

| **Plot** | **Community type** | **Elevation**  **(m a.s.l.)** | **pH** | **Topography** | **Edge (m)** | **Fragment size (ha)** | **Fragment** | **Longitude** | **Latitude** |
| --- | --- | --- | --- | --- | --- | --- | --- | --- | --- |
| T01 | Lowland | 579 | 4.92 | valley | 30 | 651.60 | 1 | 101.2702 | 21.9161 |
| T02 | Lowland | 637 | 4.77 | ridge | 200 | 651.60 | 1 | 101.2721 | 21.9173 |
| T03 | Lowland | 541 | 5.06 | midslope | 30 | 651.60 | 1 | 101.2737 | 21.9185 |
| T04 | Limestone | 680 | 5.21 | midslope | 50 | 9954.00 | 2 | 101.2827 | 21.9111 |
| T05 | Lowland | 651 | 4.80 | ridge | 20 | 63.00 | 3 | 101.2396 | 21.9194 |
| T06 | Lowland | 582 | 4.71 | midslope | 10 | 17.10 | 4 | 101.2689 | 21.8822 |
| T07 | Limestone | 1310 | 6.50 | valley | 200 | 5183.10 | 5 | 101.2283 | 21.9866 |
| T08 | Lowland | 891 | 4.31 | valley | 1047 | 138728.70 | 6 | 101.1996 | 21.9604 |
| T09 | Montane | 835 | 4.60 | ridge | 427 | 138728.70 | 6 | 101.2035 | 21.9651 |
| T10 | Montane | 1148 | 4.47 | ridge | 935 | 138728.70 | 6 | 101.1624 | 21.9732 |
| T11 | Limestone | 887 | 6.40 | valley | 254 | 138728.70 | 6 | 101.1618 | 21.9745 |
| T12 | Montane | 1205 | 4.43 | midslope | 568 | 138728.70 | 6 | 101.1633 | 21.9715 |
| T13 | Limestone | 755 | 6.22 | ridge | 300 | 9954.00 | 2 | 101.2845 | 21.9119 |
| T14 | Limestone | 727 | 7.26 | ridge | 50 | 1678.50 | 7 | 101.3671 | 21.8513 |
| T15 | Limestone | 711 | 7.20 | midslope | 20 | 0.90 | 8 | 101.3782 | 21.8830 |
| T16 | Montane | 711 | 4.42 | midslope | 310 | 549.00 | 9 | 101.2487 | 21.8922 |
| T17 | Montane | 975 | 4.58 | ridge | 110 | 17482.50 | 10 | 101.2504 | 21.8761 |
| T18 | Montane | 916 | 4.41 | ridge | 100 | 17482.50 | 10 | 101.2530 | 21.8786 |
| T19 | Montane | 844 | 3.96 | midslope | 100 | 17482.50 | 10 | 101.2548 | 21.8813 |
| T20 | Lowland | 762 | 4.24 | valley | 517 | 3298.50 | 11 | 101.2129 | 21.9136 |
| T21 | Montane | 714 | 4.64 | ridge | 313 | 3298.50 | 11 | 101.2134 | 21.9165 |
| T22 | Lowland | 663 | 4.92 | valley | 10 | 138728.70 | 6 | 101.2060 | 21.9254 |
| T23 | Montane | 733 | 4.48 | ridge | 186 | 138728.70 | 6 | 101.2083 | 21.9254 |
| T24 | Montane | 654 | 4.44 | ridge | 10 | 48.60 | 12 | 101.2763 | 21.8579 |
| T25 | Limestone | 1114 | 6.21 | midslope | 20 | 0.90 | 13 | 101.3170 | 21.8173 |
| T26 | Lowland | 734 | 4.90 | valley | 702 | 32830.20 | 14 | 101.3023 | 21.9543 |
| T27 | Lowland | 692 | 4.14 | valley | 470 | 32830.20 | 14 | 101.2983 | 21.9480 |
| T28 | Lowland | 717 | 4.46 | midslope | 490 | 32830.20 | 14 | 101.3015 | 21.9516 |
| T29 | Limestone | 678 | 4.88 | midslope | 200 | 32830.20 | 14 | 101.3218 | 21.9333 |
| T30 | Limestone | 802 | 5.56 | ridge | 485 | 32830.20 | 14 | 101.3223 | 21.9377 |
| T31 | Lowland | 619 | 6.24 | valley | 140 | 32830.20 | 14 | 101.3262 | 21.9342 |
| T32 | Limestone | 689 | 6.24 | ridge | 50 | 9954.00 | 2 | 101.2936 | 21.8954 |
| T33 | Limestone | 869 | 7.39 | valley | 20 | 9954.00 | 2 | 101.2974 | 21.8967 |
| T34 | Lowland | 620 | 6.48 | valley | 20 | 143.10 | 15 | 101.2729 | 21.8574 |
| T35 | Limestone | 1477 | 6.12 | ridge | 342 | 5183.10 | 5 | 101.2263 | 21.9869 |
| T36 | Montane | 1172 | - | midslope | 20 | 875.70 | 16 | 101.1719 | 21.9868 |
| T37 | Limestone | 994 | 7.01 | midslope | 206 | 107.10 | 17 | 101.2190 | 21.9825 |
| T38 | Lowland | 717 | 6.67 | valley | 124 | 761.40 | 18 | 101.2155 | 21.9778 |
| T40 | Montane | 849 | 4.35 | midslope | 10 | 207.00 | 19 | 101.3309 | 21.8635 |
| T41 | Montane | 894 | 4.22 | ridge | 20 | 203.40 | 20 | 101.3268 | 21.8711 |
| T42 | Montane | 1189 | 4.60 | ridge | 50 | 9258.30 | 21 | 101.3357 | 21.8287 |
| T43 | Limestone | 677 | 6.46 | midslope | 242 | 9954.00 | 2 | 101.2930 | 21.9097 |
| T44 | Limestone | 858 | 5.60 | midslope | 30 | 9954.00 | 2 | 101.3154 | 21.8647 |
| T45 | Limestone | 871 | 4.72 | ridge | 30 | 4.50 | 22 | 101.2990 | 21.8766 |
| T46 | Montane | 851 | 3.95 | ridge | 150 | 434.70 | 23 | 101.3159 | 21.8762 |
| T48 | Lowland | 733 | 4.54 | valley | 60 | 1001.07 | 24 | 101.2548 | 21.9550 |
| T49 | Lowland | 753 | 4.45 | midslope | 100 | 1001.07 | 24 | 101.2580 | 21.9530 |
| T50 | Montane | 770 | 5.41 | ridge | 20 | 1001.07 | 24 | 101.2580 | 21.9568 |

**Table S5.** Plant life-form diversity indices and density for the 48 plots.

| Plot | Trees | | | |  | Liana | | | |  | Herb | | | |  | Fern | | | |
| --- | --- | --- | --- | --- | --- | --- | --- | --- | --- | --- | --- | --- | --- | --- | --- | --- | --- | --- | --- |
|  | Richness | Density | Hill | Evenness |  | Richness | Density | Hill | Evenness |  | Richness | Density | Hill | Evenness |  | Richness | Density | Hill | Evenness |
| T01 | 40 | 3700.88 | 28.46 | 0.91 |  | 16 | 60 | 12.20 | 0.90 |  | 23 | 168 | 15.19 | 0.86 |  | 12 | 79 | 7.76 | 0.78 |
| T02 | 31 | 8997.56 | 14.60 | 0.78 |  | 19 | 87 | 12.18 | 0.85 |  | 8 | 29 | 7.25 | 0.90 |  | 7 | 46 | 4.68 | 0.70 |
| T03 | 32 | 7448.45 | 16.91 | 0.82 |  | 29 | 86 | 18.08 | 0.86 |  | 9 | 35 | 8.27 | 0.92 |  | 10 | 65 | 5.92 | 0.72 |
| T04 | 17 | 8233.62 | 5.29 | 0.59 |  | 21 | 44 | 18.25 | 0.95 |  | 10 | 27 | 6.45 | 0.78 |  | 4 | 9 | 2.73 | 0.56 |
| T05 | 37 | 7066.48 | 21.25 | 0.85 |  | 14 | 38 | 10.48 | 0.89 |  | 14 | 86 | 10.09 | 0.85 |  | 5 | 58 | 3.44 | 0.63 |
| T06 | 38 | 4456.34 | 27.87 | 0.91 |  | 24 | 58 | 17.77 | 0.91 |  | 23 | 176 | 14.72 | 0.85 |  | 6 | 23 | 4.96 | 0.77 |
| T07 | 42 | 6451.08 | 26.87 | 0.88 |  | 13 | 63 | 9.56 | 0.88 |  | 17 | 72 | 10.11 | 0.80 |  | 1 | 2 | 1.00 | 0.00 |
| T08 | 50 | 6875.49 | 33.73 | 0.90 |  | 14 | 29 | 10.66 | 0.90 |  | 20 | 84 | 12.87 | 0.84 |  | 10 | 96 | 6.29 | 0.74 |
| T09 | 21 | 9982.20 | 9.64 | 0.74 |  | 10 | 23 | 7.04 | 0.85 |  | 10 | 33 | 8.87 | 0.91 |  | 6 | 37 | 5.47 | 0.82 |
| T10 | 32 | 7712.19 | 18.42 | 0.84 |  | 7 | 14 | 6.11 | 0.93 |  | 11 | 56 | 7.75 | 0.82 |  | 4 | 18 | 3.06 | 0.62 |
| T11 | 29 | 4838.31 | 8.68 | 0.64 |  | 4 | 10 | 2.97 | 0.79 |  | 10 | 30 | 8.76 | 0.91 |  | 8 | 53 | 5.14 | 0.71 |
| T12 | 32 | 6984.63 | 15.93 | 0.80 |  | 5 | 7 | 4.37 | 0.92 |  | 9 | 27 | 6.89 | 0.84 |  | 2 | 28 | 1.29 | 0.19 |
| T13 | 10 | 6557.18 | 1.89 | 0.28 |  | 8 | 14 | 6.46 | 0.90 |  | 10 | 49 | 6.97 | 0.81 |  | 6 | 58 | 4.53 | 0.73 |
| T14 | 14 | 7275.66 | 6.28 | 0.70 |  | 13 | 37 | 9.99 | 0.90 |  | 9 | 45 | 4.25 | 0.63 |  | 1 | 2 | 1.00 | 0.00 |
| T15 | 9 | 6766.36 | 2.01 | 0.32 |  | 6 | 11 | 5.59 | 0.96 |  | 4 | 8 | 3.75 | 0.82 |  | 2 | 4 | 2.00 | 0.50 |
| T16 | 33 | 7348.41 | 20.98 | 0.87 |  | 6 | 13 | 5.08 | 0.91 |  | 10 | 56 | 6.70 | 0.79 |  | 4 | 35 | 3.59 | 0.71 |
| T17 | 22 | 7857.71 | 11.73 | 0.80 |  | 6 | 20 | 4.56 | 0.85 |  | 12 | 42 | 9.52 | 0.88 |  | 5 | 41 | 2.59 | 0.49 |
| T18 | 18 | 7202.90 | 10.54 | 0.81 |  | 5 | 23 | 3.33 | 0.75 |  | 8 | 21 | 7.30 | 0.90 |  | 4 | 27 | 2.63 | 0.54 |
| T19 | 28 | 8148.73 | 17.44 | 0.86 |  | 5 | 24 | 1.98 | 0.42 |  | 12 | 102 | 7.49 | 0.78 |  | 5 | 24 | 4.33 | 0.75 |
| T20 | 50 | 7348.41 | 39.37 | 0.94 |  | 21 | 52 | 15.50 | 0.90 |  | 19 | 113 | 10.56 | 0.79 |  | 9 | 44 | 7.26 | 0.83 |
| T21 | 20 | 8403.38 | 13.77 | 0.88 |  | 3 | 8 | 2.65 | 0.89 |  | 11 | 81 | 7.56 | 0.81 |  | 7 | 43 | 5.20 | 0.75 |
| T22 | 51 | 4037.99 | 37.75 | 0.92 |  | 21 | 54 | 9.87 | 0.75 |  | 25 | 184 | 14.79 | 0.83 |  | 10 | 61 | 7.45 | 0.81 |
| T23 | 29 | 8075.98 | 14.75 | 0.80 |  | 14 | 30 | 10.88 | 0.90 |  | 15 | 123 | 7.94 | 0.75 |  | 5 | 70 | 4.81 | 0.81 |
| T24 | 33 | 8912.68 | 20.21 | 0.86 |  | 15 | 59 | 10.17 | 0.86 |  | 15 | 75 | 12.44 | 0.91 |  | 8 | 49 | 5.75 | 0.76 |
| T25 | 30 | 6811.83 | 16.97 | 0.83 |  | 11 | 31 | 8.31 | 0.88 |  | 11 | 90 | 7.79 | 0.83 |  | 2 | 5 | 1.96 | 0.49 |
| T26 | 52 | 5092.96 | 39.36 | 0.93 |  | 15 | 47 | 9.43 | 0.83 |  | 28 | 170 | 14.85 | 0.80 |  | 9 | 57 | 5.64 | 0.72 |
| T27 | 35 | 7493.92 | 19.11 | 0.83 |  | 27 | 76 | 18.87 | 0.89 |  | 20 | 87 | 13.23 | 0.85 |  | 6 | 13 | 5.32 | 0.80 |
| T28 | 31 | 6429.86 | 10.93 | 0.70 |  | 12 | 16 | 10.95 | 0.96 |  | 17 | 66 | 11.72 | 0.85 |  | 5 | 17 | 4.08 | 0.72 |
| T29 | 18 | 5092.96 | 4.34 | 0.51 |  | 4 | 17 | 2.20 | 0.57 |  | 4 | 43 | 1.82 | 0.37 |  | 0 | 0 | 1.00 | 0.00 |
| T30 | 30 | 6748.17 | 15.56 | 0.81 |  | 24 | 55 | 16.24 | 0.88 |  | 14 | 114 | 8.98 | 0.81 |  | 5 | 12 | 4.34 | 0.75 |
| T31 | 38 | 7348.41 | 26.83 | 0.90 |  | 28 | 51 | 22.96 | 0.94 |  | 18 | 145 | 9.87 | 0.78 |  | 5 | 21 | 3.36 | 0.62 |
| T32 | 8 | 4676.26 | 2.32 | 0.40 |  | 13 | 35 | 8.96 | 0.85 |  | 3 | 8 | 2.83 | 0.75 |  | 0 | 0 | 1.00 | 0.00 |
| T33 | 11 | 4329.01 | 2.17 | 0.32 |  | 6 | 22 | 2.75 | 0.56 |  | 2 | 26 | 1.31 | 0.25 |  | 0 | 0 | 1.00 | 0.00 |
| T34 | 39 | 6684.51 | 27.49 | 0.90 |  | 24 | 71 | 14.43 | 0.84 |  | 16 | 130 | 7.38 | 0.71 |  | 6 | 41 | 4.74 | 0.75 |
| T35 | 17 | 10898.93 | 8.37 | 0.75 |  | 8 | 28 | 6.29 | 0.88 |  | 20 | 150 | 10.19 | 0.76 |  | 2 | 16 | 1.62 | 0.35 |
| T36 | 27 | 8742.91 | 16.91 | 0.86 |  | 15 | 67 | 8.58 | 0.79 |  | 16 | 91 | 12.17 | 0.88 |  | 9 | 59 | 5.80 | 0.73 |
| T37 | 24 | 6875.49 | 12.24 | 0.79 |  | 13 | 68 | 9.50 | 0.88 |  | 15 | 61 | 9.48 | 0.81 |  | 3 | 6 | 2.75 | 0.63 |
| T38 | 44 | 9082.44 | 27.03 | 0.87 |  | 26 | 59 | 19.50 | 0.91 |  | 29 | 149 | 18.83 | 0.86 |  | 15 | 101 | 11.03 | 0.85 |
| T40 | 31 | 7193.80 | 19.93 | 0.87 |  | 11 | 30 | 9.20 | 0.93 |  | 14 | 66 | 9.86 | 0.84 |  | 10 | 43 | 8.29 | 0.85 |
| T41 | 18 | 6238.87 | 9.21 | 0.77 |  | 8 | 36 | 5.83 | 0.85 |  | 6 | 27 | 4.57 | 0.78 |  | 2 | 17 | 1.44 | 0.26 |
| T42 | 38 | 5828.61 | 21.63 | 0.85 |  | 9 | 25 | 6.34 | 0.84 |  | 15 | 61 | 10.82 | 0.86 |  | 9 | 36 | 6.12 | 0.76 |
| T43 | 7 | 5715.43 | 1.69 | 0.27 |  | 7 | 11 | 6.34 | 0.95 |  | 13 | 54 | 8.34 | 0.80 |  | 2 | 9 | 1.70 | 0.38 |
| T44 | 3 | 6281.32 | 1.83 | 0.55 |  | 6 | 16 | 3.91 | 0.76 |  | 1 | 2 | 1.00 | 0.00 |  | 2 | 3 | 1.89 | 0.46 |
| T45 | 8 | 9761.50 | 1.68 | 0.25 |  | 9 | 21 | 6.75 | 0.87 |  | 10 | 54 | 5.83 | 0.74 |  | 3 | 6 | 3.00 | 0.68 |
| T46 | 19 | 8658.03 | 9.10 | 0.75 |  | 9 | 20 | 7.48 | 0.92 |  | 2 | 10 | 1.84 | 0.56 |  | 2 | 8 | 1.94 | 0.48 |
| T48 | 47 | 7066.48 | 29.40 | 0.88 |  | 22 | 69 | 15.91 | 0.90 |  | 14 | 124 | 7.25 | 0.73 |  | 11 | 67 | 6.78 | 0.75 |
| T49 | 31 | 6111.55 | 18.31 | 0.85 |  | 25 | 60 | 17.54 | 0.89 |  | 20 | 129 | 15.12 | 0.89 |  | 10 | 87 | 6.61 | 0.76 |
| T50 | 17 | 8997.56 | 6.60 | 0.67 |  | 11 | 25 | 8.25 | 0.88 |  | 12 | 79 | 8.10 | 0.82 |  | 2 | 11 | 1.80 | 0.42 |

**Table S6.** Tests for bias in tree data diversity measures caused by different numbers of sampling sub-plots. Tree diversity measures of each plot were each regressed against the sub-plot number associated with that plot. We report only the probabilities of the tests.

| Tree diversity measure | P (effect of sub-plot number) |
| --- | --- |
| Richness | NS |
| Shannon | NS |
| Fisher’s alpha | NS |
| Chao1 | NS |
| Pielou’s evenness | NS |
| Hill number | NS |

**Table S7.** Pearson correlations between diversity indices of each life-form across sampling plots. Correlations which are greater than 0.8 are highlighted.

| TREE |  |  |  |  |  |  |
| --- | --- | --- | --- | --- | --- | --- |
|  | Richness | Density | Shannon | Pielou’s evenness | Fisher’s alpha | Chao1 |
| Richness | 1 |  |  |  |  |  |
| Density | 0.209 | 1 |  |  |  |  |
| Shannon | 0.930 | 0.181 | 1 |  |  |  |
| Pielou’s evenness | 0.804 | 0.163 | 0.958 | 1 |  |  |
| Fisher’s alpha | 0.964 | 0.153 | 0.837 | 0.696 | 1 |  |
| Chao1 | 0.634 | 0.163 | 0.525 | 0.408 | 0.608 | 1 |
| Hill number | 0.962 | 0.216 | 0.909 | 0.804 | 0.964 | 0.545 |
|  |  |  |  |  |  |  |
| LIANA |  |  |  |  |  |  |
|  | Richness | Density | Shannon | Pielou’s evenness | Fisher’s alpha | Chao1 |
| Richness | 1 |  |  |  |  |  |
| Density | 0.843 | 1 |  |  |  |  |
| Shannon | 0.911 | 0.735 | 1 |  |  |  |
| Pielou’s evenness | 0.303 | 0.102 | 0.612 | 1 |  |  |
| Fisher’s alpha | 0.827 | 0.464 | 0.818 | 0.443 | 1 |  |
| Chao1 | 0.896 | 0.665 | 0.797 | 0.209 | 0.824 | 1 |
| Hill number | 0.964 | 0.745 | 0.947 | 0.458 | 0.881 | 0.836 |
|  |  |  |  |  |  |  |
| HERB |  |  |  |  |  |  |
|  | Richness | Density | Shannon | Pielou’s evenness | Fisher’s alpha | Chao1 |
| Richness | 1 |  |  |  |  |  |
| Density | 0.878 | 1 |  |  |  |  |
| Shannon | 0.859 | 0.660 | 1 |  |  |  |
| Pielou’s evenness | 0.15 | -0.080 | 0.518 | 1 |  |  |
| Fisher’s alpha | 0.922 | 0.638 | 0.874 | 0.320 | 1 |  |
| Chao1 | 0.954 | 0.870 | 0.810 | 0.109 | 0.862 | 1 |
| Hill number | 0.940 | 0.743 | 0.921 | 0.376 | 0.930 | 0.874 |
|  |  |  |  |  |  |  |
| FERN |  |  |  |  |  |  |
|  | Richness | Density | Shannon | Pielou’s evenness | Fisher’s alpha | Chao1 |
| Richness | 1 |  |  |  |  |  |
| Density | 0.877 | 1 |  |  |  |  |
| Shannon | 0.932 | 0.782 | 1 |  |  |  |
| Pielou’s evenness | 0.415 | 0.260 | 0.622 | 1 |  |  |
| Fisher’s alpha | 0.845 | 0.545 | 0.833 | 0.406 | 1 |  |
| Chao1 | 0.985 | 0.857 | 0.917 | 0.397 | 0.843 | 1 |
| Hill number | 0.969 | 0.818 | 0.95 | 0.455 | 0.868 | 0.942 |
|  |  |  |  |  |  |  |

**Table S8.** Coefficients, standard errors and importance values (IV) of parameters selected in the best model set with community type and tree diversity/ density index as predictors (with ΔAICc < 4) (see Methods section in main article for details).

| Index | Model | Liana | | |  | Herb | | |  | Fern | | |
| --- | --- | --- | --- | --- | --- | --- | --- | --- | --- | --- | --- | --- |
|  |  | Coefficient | Std. Error | IV |  | Coefficient | Std. Error | IV |  | Coefficient | Std. Error | IV |
| Richness | Intercept | 0.933 | 0.067 |  |  | 2.854 | 1.682 |  |  | 0.997 | 0.288 |  |
|  | forestlowland | 0.315 | 0.092 | 1.00 |  | 1.611 | 0.062 | 0.29 |  | 1.016 | 0.392 | 0.88 |
|  | forestmontane | -0.074 | 0.071 | 1.00 |  | -1.404 | 2.314 | 0.29 |  | 0.654 | 0.257 | 0.88 |
|  | Tree richness | 0.004 | 0.003 | 0.38 |  | 0.370 | 1.671 | 1.00 |  | 0.028 | 0.013 | 0.84 |
|  | forestlowland: Tree richness | - | - | - |  | - | - | - |  | - | - | - |
|  | forestmontane: Tree richness | - | - | - |  | - | - | - |  | - | - | - |
|  | **R2** |  |  | **0.51** |  |  |  | **0.59** |  |  |  | **0.53** |
| Density | Intercept | 22.259 | 12.618 |  |  | 1.110 | 2.311 |  |  | 2.380 | 0.929 |  |
|  | forestlowland | 27.028 | 6.376 | 1.00 |  | 15.992 | 3.424 | 1.00 |  | 4.515 | 0.688 | 1.00 |
|  | forestmontane | -5.089 | 6.704 | 1.00 |  | 3.379 | 4.930 | 1.00 |  | 2.981 | 0.702 | 1.00 |
|  | Tree density | 0.003 | 0.002 | 0.46 |  | 0.001 | 0.000 | 1.00 |  | 0.000 | 0.000 | 0.27 |
|  | forestlowland: Tree density | - | - | - |  | -0.002 | 0.001 | 1.00 |  | - | - | - |
|  | forestmontane: Tree density | - | - | - |  | -0.001 | 0.001 | 1.00 |  | - | - | - |
|  | **R2** |  |  | **0.40** |  |  |  | **0.49** |  |  |  | **0.51** |
| Hill diversity | Intercept | 2.507 | 0.320 |  |  | 1.490 | 0.224 |  |  | 1.289 | 0.278 |  |
|  | forestlowland | 1.143 | 0.591 | 1.00 |  | 0.239 | 0.285 | 0.14 |  | 0.385 | 0.602 | 0.90 |
|  | forestmontane | -0.215 | 0.446 | 1.00 |  | 0.010 | 0.217 | 0.14 |  | -0.563 | 0.963 | 0.90 |
|  | Tree Hill diversity | 0.155 | 0.124 | 0.46 |  | 0.363 | 0.062 | 1.00 |  | 0.083 | 0.116 | 0.50 |
|  | forestlowland: Tree Hill diversity | -0.362 | 0.224 | 0.11 |  | - | - | - |  | 0.155 | 0.149 | 0.88 |
|  | forestmontane: Tree Hill diversity | -0.229 | 0.279 | 0.11 |  | - | - | - |  | 0.457 | 0.186 | 0.88 |
|  | **R2** |  |  | **0.53** |  |  |  | **0.52** |  |  |  | **0.56** |
| Evenness (Pielou) | Intercept | 0.843 | 0.037 |  |  | 0.575 | 0.107 |  |  | 0.380 | 0.095 |  |
|  | forestlowland | 0.041 | 0.036 | 0.14 |  | 0.133 | 0.069 | 0.43 |  | 0.321 | 0.285 | 1.00 |
|  | forestmontane | 0.000 | 0.036 | 0.14 |  | 0.127 | 0.065 | 0.43 |  | 0.033 | 0.576 | 1.00 |
|  | Tree Evenness | 0.053 | 0.077 | 0.25 |  | 0.310 | 0.147 | 0.71 |  | 0.052 | 0.240 | 0.34 |
|  | forestlowland: Tree evenness | - | - | - |  | - | - | - |  | 0.275 | 0.869 | 0.13 |
|  | forestmontane: Tree evenness | - | - | - |  | - | - | - |  | 1.878 | 0.945 | 0.13 |
|  | **R2** |  |  | **0.04** |  |  |  | **0.19** |  |  |  | **0.41** |

**Table S9.** Coefficients, standard errors and importance values (IV) of parameters selected in the best model set (with ΔAICc < 4) with community type and plot distance to the fragment edge as predictors (see *Materials and Methods* in main article for details).

| Index | Model | Tree | | |  | Liana | | |  | Herb | | |  | Fern | | |
| --- | --- | --- | --- | --- | --- | --- | --- | --- | --- | --- | --- | --- | --- | --- | --- | --- |
|  |  | Coeff. | Std. Error | IV |  | Coeff. | Std. Error | IV |  | Coeff. | Std. Error | IV |  | Coeff. | Std. Error | IV |
| Richness | Intercept | 13.407 | 6.672 |  |  | 1.020 | 0.144 |  |  | 0.781 | 7.594 |  |  | 1.160 | 0.569 |  |
|  | forestlowland | 24.402 | 6.122 | 1.00 |  | -0.047 | 0.081 | 1.00 |  | 6.074 | 3.183 | 1.00 |  | 1.705 | 0.613 | 1.00 |
|  | forestmontane | 10.435 | 6.685 | 1.00 |  | 0.384 | 0.139 | 1.00 |  | 16.931 | 7.865 | 1.00 |  | 1.154 | 0.808 | 1.00 |
|  | Edge distance | 4.035 | 3.579 | 0.49 |  | 0.020 | 0.204 | 0.67 |  | 12.434 | 10.054 | 0.74 |  | 0.310 | 0.391 | 0.36 |
|  | forestlowland: Edge distance | -6.938 | 5.412 | 0.10 |  | -0.087 | 0.116 | 0.20 |  | -6.292 | 3.092 | 0.74 |  | -0.611 | 0.414 | 0.17 |
|  | forestmontane: Edge distance | -8.202 | 5.546 | 0.10 |  | -0.209 | 0.119 | 0.20 |  | -9.028 | 3.169 | 0.61 |  | -0.931 | 0.424 | 0.17 |
| Density | Intercept | 6410 | 745 |  |  | 24.706 | 20.684 |  |  | 2.791 | 3.659 |  |  | 1.453 | 2.143 |  |
|  | forestlowland | -135 | 529 | 0.80 |  | 3.193 | 10.975 | 1.00 |  | 9.054 | 4.801 | 1.00 |  | 5.676 | 2.488 | 1.00 |
|  | forestmontane | 1185 | 529 | 0.80 |  | 47.719 | 24.293 | 1.00 |  | 5.55 | 4.582 | 1.00 |  | 4.197 | 2.505 | 1.00 |
|  | Edge distance | 463 | 368 | 0.40 |  | 28.723 | 32.003 | 0.88 |  | 2.821 | 1.566 | 0.70 |  | 1.361 | 1.241 | 0.44 |
|  | forestlowland: Edge distance | - | - | - |  | -17.692 | 10.658 | 0.59 |  | -4.279 | 1.52 | 0.61 |  | -2.511 | 1.202 | 0.24 |
|  | forestmontane: Edge distance | - | - | - |  | -27.75 | 10.922 | 0.59 |  | -3.991 | 1.558 | 0.61 |  | -2.524 | 1.231 | 0.24 |
| Hill diversity | Intercept | 2.351 | 0.374 |  |  | 2.747 | 0.250 |  |  | 0.863 | 0.798 |  |  | 1.399 | 0.274 |  |
|  | forestlowland | 2.589 | 0.335 | 1.00 |  | 1.129 | 0.223 | 1.00 |  | 2.463 | 0.873 | 1.00 |  | 1.016 | 0.296 | 1.00 |
|  | forestmontane | 1.343 | 0.335 | 1.00 |  | -0.214 | 0.223 | 1.00 |  | 2.408 | 1.039 | 1.00 |  | 0.585 | 0.395 | 1.00 |
|  | Edge distance | 0.179 | 0.232 | 0.28 |  | -0.120 | 0.155 | 0.28 |  | 0.887 | 0.282 | 0.86 |  | 0.096 | 0.219 | 0.86 |
|  | forestlowland: Edge distance | - | - | - |  | - | - | - |  | -0.828 | 0.352 | 0.86 |  | -0.313 | 0.283 | 0.86 |
|  | forestmontane: Edge distance | - | - | - |  | - | - | - |  | -1.162 | 0.361 | 0.86 |  | -0.546 | 0.290 | 0.86 |
| Evenness | Intercept | 0.518 | 0.102 |  |  | 0.839 | 0.038 |  |  | 0.576 | 0.174 |  |  | 0.381 | 0.074 |  |
|  | forestlowland | 0.344 | 0.118 | 1.00 |  | 0.041 | 0.036 | 0.13 |  | 0.237 | 0.187 | 1.00 |  | 0.357 | 0.074 | 1.00 |
|  | forestmontane | 0.285 | 0.109 | 1.00 |  | 0.000 | 0.036 | 0.13 |  | 0.24 | 0.206 | 1.00 |  | 0.231 | 0.074 | 1.00 |
|  | Edge distance | 0.056 | 0.07 | 0.34 |  | 0.024 | 0.025 | 0.30 |  | 0.111 | 0.097 | 0.47 |  | 0.02 | 0.052 | 0.24 |
|  | forestlowland: Edge distance | -0.154 | 0.088 | 0.10 |  | - | - | - |  | -0.183 | 0.097 | 0.24 |  | - | - | - |
|  | forestmontane: Edge distance | -0.133 | 0.09 | 0.10 |  | - | - | - |  | -0.209 | 0.099 | 0.24 |  | - | - | - |

**Table S10.**  Coefficients, standard errors and importance values (IV) of parameters selected in the best model set (with ΔAICc < 4) with community type and fragment size where each plot is located as predictors (see *Materials and Methods* in main article for details).

| Index | Model | Tree | | |  | Liana | | |  | Herb | | |  | Fern | | |
| --- | --- | --- | --- | --- | --- | --- | --- | --- | --- | --- | --- | --- | --- | --- | --- | --- |
|  |  | Coeff. | Std. Error | IV |  | Coeff. | Std. Error | IV |  | Coeff. | Std. Error | IV |  | Coeff. | Std. Error | IV |
| Richness | Intercept | 15.357 | 4.886 |  |  | 1.013 | 0.116 |  |  | 8.91 | 2.503 |  |  | 1.401 | 0.278 |  |
|  | forestlowland | 23.004 | 3.047 | 1.00 |  | 0.351 | 0.067 | 1.00 |  | 9.355 | 1.835 | 1.00 |  | 1.498 | 0.235 | 1.00 |
|  | forestmontane | 8.675 | 3.06 | 1.00 |  | -0.057 | 0.067 | 1.00 |  | 1.517 | 1.84 | 1.00 |  | 0.839 | 0.236 | 1.00 |
|  | Fragment size | 0.952 | 0.949 | 0.33 |  | -0.024 | 0.021 | 0.37 |  | 0.39 | 0.574 | 0.27 |  | -0.016 | 0.074 | 0.23 |
|  | forestlowland: Fragment size | - | - | - |  | - | - | - |  | - | - | - |  | - | - | - |
|  | forestmontane: Fragment size | - | - | - |  | - | - | - |  | - | - | - |  | - | - | - |
| Density | Intercept | 7200.9 | 915.1 |  |  | 40.046 | 15.594 |  |  | 6.663 | 1.046 |  |  | 2.433 | 0.898 |  |
|  | forestlowland | -114.0 | 529.8 | 0.87 |  | 33.275 | 19.347 | 1.00 |  | 3.893 | 0.9 | 1.00 |  | 4.504 | 0.688 | 1.00 |
|  | forestmontane | 1222.3 | 532.9 | 0.87 |  | 4.442 | 21.849 | 1.00 |  | 0.752 | 0.902 | 1.00 |  | 3.012 | 0.69 | 1.00 |
|  | Fragment size | -206.9 | 164.3 | 0.35 |  | -2.495 | 2.613 | 0.64 |  | -0.004 | 0.283 | 0.22 |  | 0.123 | 0.216 | 0.26 |
|  | forestlowland: Fragment size | - | - | - |  | -6.018 | 4.691 | 0.16 |  | - | - | - |  | - | - | - |
|  | forestmontane: Fragment size | - | - | - |  | -7.05 | 4.782 | 0.16 |  | - | - | - |  | - | - | - |
| Hill diversity | Intercept | 2.413 | 0.399 |  |  | 2.862 | 0.387 |  |  | 2.349 | 0.251 |  |  | 1.458 | 0.184 |  |
|  | forestlowland | 2.589 | 0.336 | 1.00 |  | 1.134 | 0.223 | 1.00 |  | 1.062 | 0.213 | 1.00 |  | 0.957 | 0.159 | 1.00 |
|  | forestmontane | 1.341 | 0.336 | 1.00 |  | -0.201 | 0.224 | 1.00 |  | 0.436 | 0.214 | 1.00 |  | 0.482 | 0.159 | 1.00 |
|  | Fragment size | 0.027 | 0.105 | 0.23 |  | -0.08 | 0.069 | 0.37 |  | 0.012 | 0.067 | 0.23 |  | -0.002 | 0.05 | 0.22 |
|  | forestlowland: Fragment size | - | - | - |  | - | - | - |  | - | - | - |  | - | - | - |
|  | forestmontane: Fragment size | - | - | - |  | - | - | - |  | - | - | - |  | - | - | - |
| Evenness | Intercept | 0.552 | 0.058 |  |  | 0.908 | 0.076 |  |  | 0.71 | 0.085 |  |  | 0.423 | 0.107 |  |
|  | forestlowland | 0.313 | 0.049 | 1.00 |  | 0.042 | 0.036 | 0.18 |  | 0.153 | 0.056 | 1.00 |  | 0.358 | 0.074 | 1.00 |
|  | forestmontane | 0.258 | 0.049 | 1.00 |  | 0.004 | 0.036 | 0.18 |  | 0.145 | 0.056 | 1.00 |  | 0.234 | 0.075 | 1.00 |
|  | Fragment size | 0.003 | 0.015 | 0.23 |  | -0.017 | 0.011 | 0.51 |  | -0.016 | 0.017 | 0.31 |  | -0.018 | 0.023 | 0.29 |
|  | forestlowland: Fragment size | - | - | - |  | - | - | - |  | - | - | - |  | - | - | - |
|  | forestmontane: Fragment size | - | - | - |  | - | - | - |  | - | - | - |  | - | - | - |
